# Decoupling of motor cortex to movement in Parkinson’s dyskinesia rescued by sub-anaesthetic ketamine

**DOI:** 10.1093/brain/awae386

**Published:** 2024-11-25

**Authors:** Abhilasha Vishwanath, Mitchell J Bartlett, Torsten Falk, Stephen L Cowen

**Affiliations:** Department of Psychology, The University of Arizona, Tucson, AZ 85721, USA; Department of Surgery, The University of Arizona, Tucson, AZ 85724, USA; Department of Neurosurgery, The University of Arizona, Tucson, AZ 85724, USA; Department of Neurology, The University of Arizona, Tucson, AZ 85724, USA; Department of Neurology, The University of Arizona, Tucson, AZ 85724, USA; Department of Pharmacology, The University of Arizona, Tucson, AZ 85724, USA; Department of Psychology, The University of Arizona, Tucson, AZ 85721, USA

**Keywords:** *in vivo* electrophysiology, population state, inertial speed, cell types, 6-hydroxydopamine hemi-lesioned model

## Abstract

Gamma-band and single-unit neural activity in primary motor cortex are involved in the control of movement. This activity is disrupted in Parkinson’s disease (PD) and levodopa-induced dyskinesia (LID), a debilitating consequence of dopamine replacement therapy for PD. Physiological features of LID include pathological narrowband gamma oscillations, finely tuned gamma and altered primary motor cortex firing activity. Given that most studies characterize LID through visual scoring, little is known about the relationships between ongoing dyskinetic movements, gamma and neuronal activity at fast (sub-second) and slow (seconds) time scales. Here, we investigate how motor cortex activity changes with movement at multiple time scales in animal models of PD and LID. Furthermore, sub-anaesthetic ketamine has emerged as a possible therapy for LID. How ketamine might reduce LID is not fully understood. Consequently, we investigate how ketamine affects the relationship between motor cortex activity and movement.

To investigate these questions, local-field and single-unit activity from >3000 motor cortex neurons was acquired using a standard model of PD/LID (*n* = 10 male rats). Data in LID and sham animals were acquired following levodopa (L-DOPA; 12 mg/kg, intraperitoneal) and ketamine (20 mg/kg, intraperitoneal) administration. Movement was assessed using traditional abnormal involuntary movement scores and head-mounted inertial sensors sampled at 200 Hz.

Although correlations between movement, gamma and single-unit activity were high in all animals during control conditions, correlations decreased considerably in animal models of LID following L-DOPA administration. This suggests that primary motor cortex can become functionally decoupled from ongoing movements in LID. Interestingly, this effect was observed in both the dopamine-depleted and non-depleted hemispheres. Ketamine disrupted finely tuned gamma, decreased LID and moderately increased single-unit correlations with movement during LID. Ketamine, however, did not enhance the correlation between gamma-band activity and movement. Finally, ketamine exerted a selective effect on neuronal interactions and ensemble activity in LID animals. Specifically, analysis of cell-pair firing-rate correlations showed that ketamine induced a distinct neural ensemble state in LID by reorganizing the pattern of cell-pair interactions.

These findings provide insight into the role that motor cortex neurons and gamma-band activity play during healthy movement and LID. Results suggest that primary motor cortex does not directly trigger specific dyskinetic movements during LID but, instead, dysregulated motor cortex activity might permit aberrant movements to emerge spontaneously in downstream circuits. These data further support the anti-dyskinetic properties of ketamine and suggest that ketamine acts to reduce LID by disrupting pathological interactions between motor cortex neurons during dyskinesia.

## Teaser

Vishwanath *et al.* have studied the relationship between neural activity in motor cortex and the abnormal movements associated with dyskinesia in Parkinson's disease. In rats, dyskinesia was marked by decoupling of neural activity from ongoing movement. Sub-anaesthetic doses of ketamine restored this coupling and reduced dyskinesia.

## Introduction

Parkinson’s disease (PD) is associated with debilitating motor and cognitive deficits resulting from the degeneration of midbrain dopaminergic neurons. Although levodopa (L-DOPA) has been the gold-standard treatment for >60 years,^1^ nearly 40% of PD patients develop L-DOPA-induced dyskinesia (LID) after 4–6 years.^2^ The excessive uncontrolled movements associated with LID are debilitating, and current treatments are limited.

Chronic dopamine depletion in PD alters the cortico-basal ganglia–thalamic circuit.^3-7^ The motor cortex is a key component of this circuit because it influences the activity of basal ganglia neurons,^8-10^ receives strong input for motor thalamus, and regulates action selection and initiation.^11-13^ Moreover, neuro-adaptations of the basal ganglia in PD alter primary motor cortex (M1) excitability,^14-17^ synchrony,^18-20^ and the relationship between M1 activity and movement.^21,22^

M1 activity is also strongly affected by LID. For example, ∼80 Hz finely tuned gamma (FTG) oscillations in M1 and basal ganglia subregions are a signature of LID.^7,23-26^ In addition, LID is associated with increased glutamate transmission and excitability of M1 neurons.^27-29^ Despite the characterization of these changes in LID, the relationship between ongoing M1 single-unit and local-field activity neurons and ongoing movements during LID is not well understood. Here, we investigate the relationship between single-unit and local-field activity and movement during LID.

Ketamine is a dissociative anaesthetic and *N*-methyl-D-aspartate (NMDA) receptor antagonist with additional biological targets.^30^ Sub-anaesthetic ketamine also acts as a potent antidepressant.^31,32^ Previous work by our group has shown that repeated administration of sub-anaesthetic ketamine reduces established LID for weeks,^33^ eliminates oscillatory signatures of LID in M1 and striatum,^34^ attenuates the development of LID^35^ and reduces mushroom spine density in striatal medium spiny neurons, which are highly correlated with LID.^36^ A clinical case study further supported anti-dyskinetic activity of ketamine,^37^ and in a Phase 1 clinical trial (NCT06021756) both safety and tolerability were shown, and possible efficacy in individuals with LID was evident.

Ketamine has a variety of effects on cortical single-unit activity. Low-to-moderate doses increase the activity of cortical neurons^38-41^ and decrease burst-firing.^42,43^ It has been hypothesized that this results from ketamine preferentially binding to NMDA receptors expressed on parvalbumin-expressing (PV) interneurons,^40,44,45^ resulting in the disinhibition of cortical principal cells. Although compelling, this model has not been examined rigorously and is not entirely consistent with the observation that ketamine triggers strong M1 gamma,^46-48^ an oscillation thought to involve the activation rather than inhibition of PV neurons.^49-51^

To examine the role of M1 in LID and in ketamine-mediated attenuation of LID, we measured M1 single-unit and local-field activity in LID following ketamine administration. These investigations identified a notable decoupling between M1 gamma-band activity and neural firing to ongoing movements during dyskinesia. Interestingly, despite ketamine clearly reducing LID, it did not increase or decrease the correlations between gamma and movement during LID. Ketamine did, however, moderately increase the correlation between single-unit activity and movement during LID. Finally, we observed that M1 cell-pair interactions were largely unaffected by LID but were affected by ketamine in dyskinetic animals. Specifically, we observed that ketamine reconfigured the arrangement of cell-pair correlations without systematically increasing or decreasing their values.

## Materials and methods

### Animals

Ten male Sprague–Dawley rats (230–300 g on arrival; Envigo) were used for the study, out of a total 15 rats. Animals were housed in a 12 h reverse day/night cycle room for the duration of the study. Food and water were provided *ad libitum*, except for one rat that was food restricted to motivate performance on a motor task (data not shown). All procedures were in accordance with National Institutes of Health guidelines for the Care and Use of Laboratory Animals and the ARRIVE guidelines.

### Surgical procedures

#### Preclinical model of established LID

**Figure 1:**
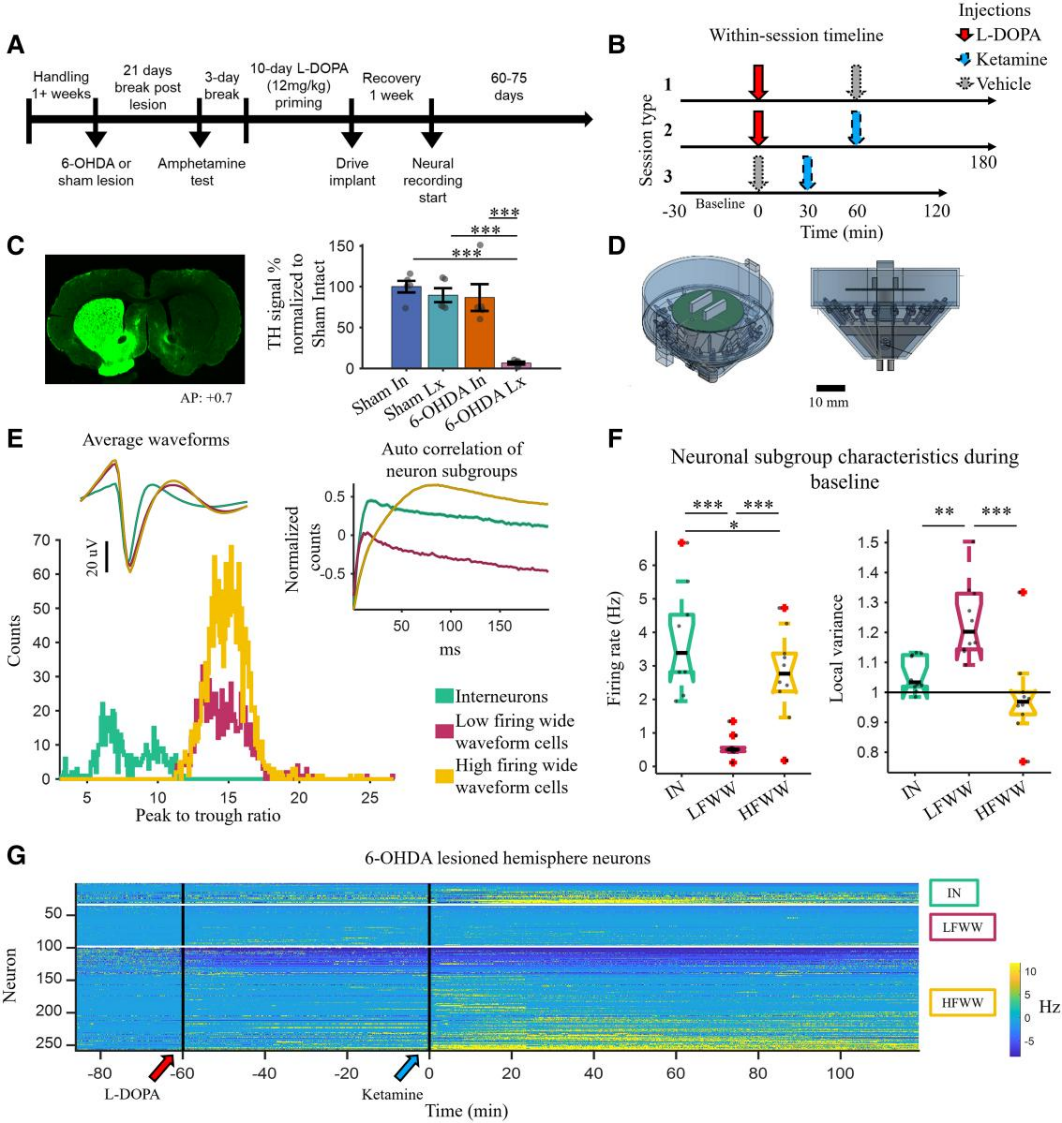
**Experimental conditions and lesion confirmation.** (**A**) Schematic diagram of the experimental time line. Animals underwent a 6-hydroxydopamine (6-OHDA) lesion or sham lesion procedures after a handling period. Lesions were confirmed using an amphetamine test 21 days after the lesion procedure. Animals were treated with levodopa (L-DOPA; 12 mg/kg) for 10 consecutive days to establish dyskinesia. Animals that showed dyskinesia (see ‘Materials and methods’ section for criteria) were implanted with dual-bundle hyperdrives, and after recovery neural recording was conducted every 3–5 days for 60–75 days. (**B**) There were three types of recording sessions with different drug combinations: L-DOPA followed by vehicle or ketamine (20 mg/kg) at 60 min post L-DOPA injection (1 and 2), and vehicle followed by ketamine at 30 min post vehicle injection (3). The vehicle injections served as a control for either ketamine or L-DOPA, and a control for changes related to injection trauma. (**C**) *Left*: Tyrosine hydroxylase (TH)-stained tissue slice from one representative 6-OHDA-lesioned animal. *Right*: Percentage TH signal normalized to the sham-intact hemisphere, where the 6-OHDA-lesioned hemisphere showed significant decrease in TH compared with controls [ANOVA, *F*(3,16) = 18.84, *P* < 0.001, η^2^ = 0.7794]. (**D**) High-density dual-bundle 16-tetrode hyperdrive designs custom made for the experiment. (**E**) Peak-to-trough ratio of the three classified neuron subgroups [interneurons, low-firing wide-waveform (LFWW) cells and high-firing wide-waveform (HFWW) cells], showing a distinction between narrow and wide waveforms. *Inset right*: Autocorrelation for each of the three subgroups. This was used along with the firing rate of the wide waveform neurons to classify LFWW and HFWW cells. *Inset left*: Plot showing the average waveform of all neurons in the three subgroups. (**F**) *Left*: Baseline firing rate for each animal (*n* = 10, 5 per Sham and levodopa-induced dyskinesia groups) of the classified neuron subgroups shown in **E**. The firing rates of principal cells were significantly lower than those of interneurons and HFWW cells (*P* = 0.0001 and *P* = 0.0002, respectively, *t*-test with Bonferroni–Holm correction). The interneurons had a significantly higher firing rate than the HFWW cells (*P* = 0.0293). *Right*: Baseline local variance of neuronal subgroups across animals (*n* = 10, 5 per sham and 6-OHDA groups). The LFWW cells had a significantly higher local variance than the interneurons and the HFWW cells (*P* = 0.0011 and *P* = 0.0007, respectively, *t*-test with Bonferroni–Holm correction). (**G**) Baseline normalized firing rate of all recorded neurons from the 6-OHDA-lesioned hemisphere binned at 500 ms. Neurons sorted by firing rate. *P < 0.05, **P < 0.01 and ***P < 0.001. Error bars show the mean ± standard error of the mean.

Unilateral 6-hydroxydopamine (6-OHDA) lesions were performed as published^36^ and with the following modifications (Fig. 1A). A total of 25 µg of 6-OHDA was injected into the medial forebrain bundle (12.5 µg in 2.5 µl per site) at these coordinates (anteroposterior, −1.8 mm; mediolateral, +2.0 mm; dorsoventral, −8.2 mm; and anteroposterior, −2.8 mm; mediolateral, +1.8 mm; dorsoventral, −8.2 mm). Sham-treated animals were injected with the same volume of 0.02% ascorbic acid. Lesions were verified using an amphetamine (5 mg/kg, intraperitoneal; Sigma-Aldrich) rotation test, as described previously,^36^ with an inclusion criteria of ≥5 net ipsilateral rotations/min for 6-OHDA-lesioned animals and <1 net ipsilateral rotation/min for sham-lesioned animals. Sham and 6-OHDA animals were then treated with L-DOPA/benserazide (12/15 mg/kg, intraperitoneal; Sigma-Aldrich) for 10 days consecutively to establish LID.

##### Abnormal involuntary movements rating scale

LID severity was determined by a blinded experimenter using the abnormal involuntary movements (AIMs) rating scale described by Dekundy *et al*.^52^ and established in our laboratory.^53-55^ 6-OHDA-treated animals with a cumulative AIMs score >30 were included in the LID group. The cumulative limb, axial and orolingual (LAO) score at baseline for the LID group was 63.4 ± 8.14 (*n* = 5). Sham animals (*n* = 5) displayed no dyskinesia (LAO score was zero). Groups received a maintenance dose of L-DOPA every 3–5 days for the remainder of the experiment.

#### Hyperdrive implantation

Animals were anaesthetized with isoflurane and stereotaxically implanted^48^ with 16-tetrode dual-bundle (eight tetrodes per bundle) hyperdrives (Neurotek IT Inc.; Fig. 1D). Tetrodes were made from polyamide-coated 13 µm nichrome wire. Bundles were implanted over M1 (anteroposterior, 1.5 mm; mediolateral, ±2.2 mm; dorsoventral, 0.5–2.1 mm), with each bundle targeting one hemisphere, primarily over the neck/whisker^56,57^ and caudal forelimb areas^58,59^ (Supplementary Figs 1 and 2). Some tetrodes were lowered to the dorsomedial striatum (dorsoventral, 2.8–4 mm; data not shown). A skull screw over the cerebellum served as ground and reference. Animals recovered for 1 week before starting neural recordings.

### Neural data acquisition

Neural data were acquired using the Intan RHD recording system (Intan Technologies LLC) at 30 kHz.

A unique subset of neurons was obtained by moving tetrodes deeper ≥12 h before each recording session, to reduce instability. Recordings were conducted every 3–5 days. During sessions when L-DOPA was administered, LAO-AIMs were scored every 20 min for ≥180 min after injection.

#### Session types and groups

Neural activity in LID animals (*n* = 5) was acquired from the 6-OHDA-lesioned and intact hemispheres in 6-OHDA animals, and in sham animals (*n* = 5) from the sham-lesioned and intact hemispheres. All animals were exposed to three drug conditions with vehicle controls (Fig. 1B). Five sessions were recorded, on average, in each drug condition per animal.

The sample size was determined *a priori* using one of the primary outcomes: FTG power following ketamine compared with vehicle in LID animals. We estimated ketamine to have a ≥80% reduction in FTG power based on previous work from our laboratory.^34^ FTG power during dyskinesia with vehicle control was estimated at 0.0286 with 0.0121 standard deviation (SD) (pilot data). Power analysis (sampsizepwr, MATLAB), using two-tailed *t*-test, α = 0.05 and Power (1 − false negative likelihood) = 0.8, yielded a sample size of five.

### Inertial measurement

The Intan headstage has an integrated three-axis inertial measurement unit that allows the acquisition of high-resolution head and body movement data. Inertial data were acquired at 30 kHz and downsampled to 200 Hz. Motion was quantified as ‘inertial speed’, computed as the absolute value of the first temporal derivative and averaged across the six dimensions (*x* and *y* in each axis).

### Tetrode and lesion confirmation

Tetrode locations were confirmed using Cresyl Violet staining^48^ (Supplementary Fig. 1). Lesions were confirmed using infrared immunostaining for tyrosine hydroxylase (TH; Fig. 1C).^60,61^ Briefly, striatum sections were incubated in a blocking buffer containing Odyssey Intercept® (TBS) blocking buffer (OIBB; LI-COR Biosciences) and 10% normal donkey serum (Jackson ImmunoResearch Laboratories Inc.) for 1 h at room temperature. Sections were then incubated in OIBB, with the addition of 0.1% Tween-20, for TH (1:2000; Millipore) for 16 h at 4°C. TH was labelled with the secondary fluorophore IRDye® 800 CW donkey anti-rabbit (1:200; LI-COR Biosciences) in OIBB with 0.1% Tween-20 for 1 h. TH immunoreactivity was determined using Image Studio Lite (LI-COR Biosciences) to quantify signal intensity, as published previously.^62^

### Pre-processing

#### Measuring spectral activity

Local-field activity was analysed from individual tetrode channels selected based on signal quality through: (i) visual inspection of the data, to identify channels with electrical artefacts or 60 Hz noise; and (ii) evaluation of a signal-to-noise measure indicating overall gamma-band power (excluding 60 Hz). Data from the selected channel were downsampled to 500 Hz prior to analysis. Spectrograms were computed using short-time Fourier transform (spectrogram, MATLAB) for frequencies between 1 and 120 Hz with a 1 s window (500 ms overlap). The irregular-resampling auto-spectral analysis (IRASA) method for power spectra estimation^63^ was also used for the targeted analysis of the oscillatory component of the local-field signal.

#### Spike sorting

Action potentials were detected by filtering the signal (600–7000 Hz) and applying a threshold of 5 SD. Single units were identified using features of the action potential waveform using standard methods.^64^ The KlustaKwik algorithm^65^ was used as an initial step for identifying spikes. Clusters were then manually sorted, merged or split using MClust v.4.4 (https://github.com/adredish/MClust-Spike-Sorting-Toolbox).

### Analysis

#### Classification of neuronal subtypes

The criteria for inclusion of a neuron in analysis were as follows: (i) no peaks within the 2 ms window of the autocorrelogram; and (ii) firing rate of >0.1 Hz during the 20 min baseline period or the post-drug periods. A total of 3633 M1 neurons from 10 rats (363.3 ± 64.47 neurons per animal) collected over 145 recording sessions met criteria and were used for further analysis (377.6 ± 124.41 neurons per 6-OHDA-lesioned animal, *n* = 5; and 412.6 ± 71.16 neurons per sham animal, *n* = 5). Although tetrodes were moved frequently to acquire new neurons, a small subset of the 3633 neurons are likely to be repeated measures of the same neuron across sessions.

Motor cortex neurons were classified into three groups based on waveform shape using a modified version of methods previously described.^66,67^ Initially, neurons were separated into two subgroups using k-means clustering based on peak-to-trough and half-width ratios (Fig. 1E). The narrow waveform cluster was classified as putative interneurons (IN, *n* = 571). The wide waveform cluster was segmented further based on the lag time of the peak in the autocorrelogram (Fig. 1E, inset right), which indicated firing activity and temporal firing statistics. These two subgroups were labelled as low firing wide waveform (LFWW, *n* = 1061) and high-firing wide-waveform (HFWW, *n* = 2001) cells. This approach was taken instead of labelling all wide-waveform cells as putative principal cells, because cells in the HFWW group had waveform shapes similar to principal cells but firing rates approaching those of interneurons. The three groups had distinct firing rates [Fig. 1F; *n* = 10, ANOVA: *F*(2,27) = 19.3, *P* < 0.001, *η*^2^ = 0.5884]. Baseline normalized firing rates of all 6-OHDA-lesioned hemisphere neurons are shown in Fig. 1G.

M1 neurons were also divided by cortical layers based on the estimated depths of neurons during recording sessions. We defined three layers in M1: L23 (<750 µm), L5 (750–1400 µM) and L6 (1400–2100 µm). The number of neurons by layer and their firing rates are shown in Supplementary Fig. 7.

#### Measurement of burst activity

The temporal structure of the firing activity of individual neurons was quantified using ‘local variance’ (LV), a measure that assesses the variability of inter-spike intervals (ISIs) and is robust to changes in firing rate.^68^ The LV is computed from the vector of ISIs for each neuron as shown in the formula below:


(1) $$\mathrm{LV}=\frac{3}{n-1}\sum_{i=1}^{n-1}\left(1+\frac{4I_{i}I_{i+1}}{{\left(I_{i}+I_{i+1}\right)}^{2}}\right)\left(1-\frac{4R}{I_{i}+I_{i+1}}\right)\;$$

where $I_{i}$ and $I_{i+1}$ are the *i*th and i+1th ISI, and *n* is the number of ISIs. LV scores close to zero indicate tonic firing, scores close to one show Poisson firing, and scores higher than one indicate burst-like firing patterns. The LV for the neuronal subgroups during baseline conditions is shown in Fig. 1F, with strong differences between cell types [ANOVA: *F*(2,27) = 12.53, *P* = 0.0001, *η*^2^ = 0.4813]. The LFWW cells had a mean LV higher than one, indicating a burst-like firing pattern, and LFWW cells had significantly higher LV than the interneurons and HFWW cells (*P* = 0.0052 and *P* < 0.001, respectively), further suggesting that the three neuron types represent distinct cell classes.

#### Relating spectral activity to movement

To measure the relationship between spectral activity and inertial speed, the spectrogram was obtained (spectrogram, MATLAB, 1–120 Hz, 0.5 Hz increments). Power values at each frequency were converted to *z*-scores, and the Pearson’s correlation between power in each frequency and inertial speed were computed (corr, MATLAB) at 0.5 s resolution throughout a 20 min period for the following conditions: vehicle (5–25 min following vehicle injection); ketamine (5–25 min following ketamine injection); L-DOPA (65–85 min following L-DOPA injection); and L-DOPA followed by ketamine (5–25 min following ketamine injection).

To explore the overall relationship between spectral power and motion further, two regression models were generated using separate regions of the spectrogram [beta-band (15–30 Hz) and gamma-band (>35 Hz)], with inertial speed as the outcome variable. The adjusted *R*^2^ values of these models indicated the contribution of these frequency bands to movement. The intermediate 30–35 Hz range was not analysed.

#### Relating single-unit activity to movement

To quantify the relationship between single-unit activity and motion, the firing rate measured at different time scales was correlated with inertial speed. The analysis was performed across time scales to limit assumptions regarding the optimal time scale by which M1 single-unit activity responds to movement. This was accomplished by initally ‘binning’ spike times using 5 ms bins. This firing-rate vector was smoothed using a moving average (movmean, MATLAB) using the following window sizes: 50, 100, 150, 200, 250, 300, 400, 500, 800, 900, 1000, 1250, 1500, 2000, 2500, 3000, 3500 and 4000 ms. The smoothed vector was correlated with inertial speed to yield a measure of the relationship between movement and firing activity at each time scale.

#### Comparing neural ensemble states

To measure the extent to which LID and ketamine altered the ensemble state of neurons, we derived a state similarity measure that compared the pattern of pairwise correlations between neurons in two states. This measure was similar to the explained variance (EV) measure used to assess memory-trace reactivation during sleep.^69^ State similarity was computed as follows. First, neural activity for each neuron in a given session and condition was binned (1 s). Second, Pearson’s correlations between all pairwise neuron combinations were computed (corr, MATLAB). The vector of correlations in one state was then compared with the vector for a different state using Pearson’s *R*. The resulting correlation indicated the global similarity in the pattern of between-neuron firing activity between two states.

#### Statistics

Inferential statistics were calculated using MATLAB (R2023b). Analyses involved parametric tests (*t*-tests or ANOVA with Bonferroni–Holm or Tukey’s honestly significant difference correction) and non-parametric tests (Wilcoxon signed rank or Kruskal–Wallis with Bonferroni–Holm or Dunn–Sidak correction) depending on the distribution of the data. For example, non-parametric tests were used for firing rate and cell-pair correlation data, given their non-normal distributions (Shapiro–Wilk *P* < 0.05). A two-way ANOVA with Tukey’s honestly significant difference correction was used when comparing hemispheres/groups and drug conditions. Alpha was set to 0.05.

## Results

### Ketamine reduces levodopa-induced dyskinesia but does not affect overall movement speed in dyskinetic rats

**Figure 2:**
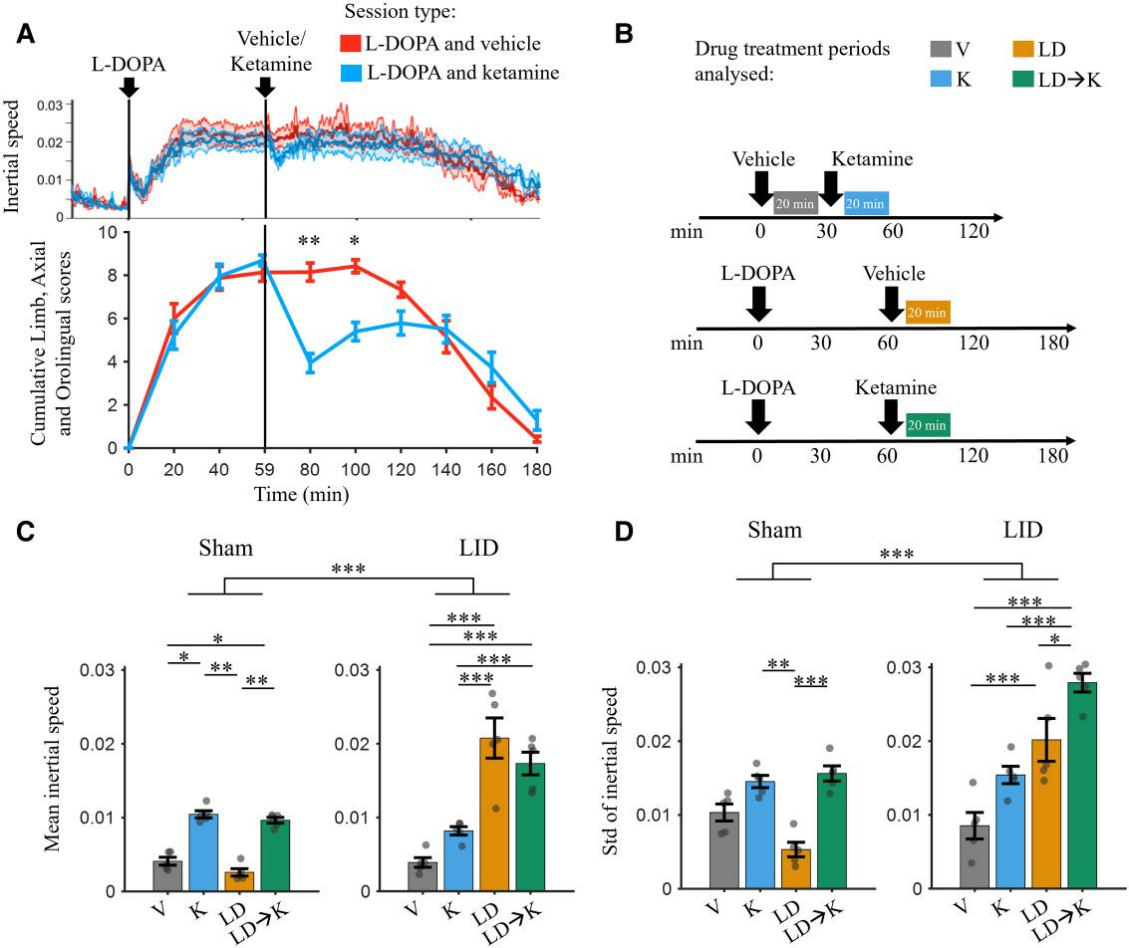
**Ketamine-induced reduction of dyskinetic behaviour and characteristics of movement speed.** (**A**) Inertial speed (*top*) and limb, axial and orolingual (LAO) scores (*bottom*) (*n* = 5, LID animals) for the levodopa (L-DOPA) followed by vehicle session type and the L-DOPA followed by ketamine session type. LAO was scored at 20 min intervals. L-DOPA injection was given at 0 min, and either vehicle or ketamine injection was given at 60 min post L-DOPA in separate sessions. LAO scores reduced significantly post ketamine when compared with post vehicle for up to 40 min (80 min, *P* = 0.0093; 100 min, *P* = 0.033, *t*-test). Shaded regions show the 95% confidence intervals (*top*). (**B**) Schematic diagram of session types (*row*) and the 20 min post-drug treatment periods used for analysis (coloured rectangles). V = vehicle (5–25 min following vehicle injection); K = ketamine (5–25 min following ketamine injection); LD = L-DOPA (65–85 min following L-DOPA injection); LD → K = L-DOPA followed by ketamine (5–25 min following ketamine injection). (**C**) Mean inertial speed for the Sham (*n* = 5) and LID animals (*n* = 5) during the drug conditions in **B**. There was a significant effect of drug condition [two-way ANOVA: *F*(3,32) = 23.65, *P* < 0.001] and group [*F*(1,32) = 47.75, *P* < 0.001] on inertial speed and an interaction effect [*F*(3,32) = 29.97, *P* < 0.001]. (**D**) Same as in **C** but for standard deviation of inertial speed. A two-way ANOVA revealed a significant effect of drug condition [two-way ANOVA: *F*(3,32) = 23.31, *P* < 0.001] and group [*F*(1,32) = 36.75, *P* < 0.001] on inertial variability, in addition to an interaction effect [*F*(3,32) = 14.55, *P* < 0.001]. *P < 0.05, **P < 0.01 and ***P < 0.001. Error bars show the mean ± standard error of the mean. LID = levodopa induced dyskinesia.

Sub-anaesthetic ketamine has been shown to reduce LAO scores in the 6-OHDA hemi-lesioned rat LID model. This effect has been observed during the ∼1 h following ketamine injection (20 mg/kg, intraperitoneal)^36^ and when assessed 30 days later.^33^ The present analysis focused on the acute effects of ketamine on LID. We found that acute administration replicated previous results, showing that ketamine (20 mg/kg, intraperitoneal) reduced LAO scores for ≥40 min (Fig. 2A; *P*_80min_ = 0.0093 and *P*_100min_ = 0.033, *t*-test).

Although LAO-AIMs scoring is the standard for quantifying the severity of dyskinetic behaviour,^52,70,71^ only a few studies have characterized other movement features, such as movement velocity and movement variability.^72-74^ Furthermore, LAO scores in preclinical models are typically acquired every 15–20 min. To assess fast changes in movement, we analysed the effects of LID acquired from head-mounted inertial sensors sampled at 200 Hz. Two measures were assessed: inertial speed (absolute value of first derivative of the inertial signal) and inertial variability (standard deviation of the inertial speed). There was a significant correlation between mean inertial speed and LAO scores in LID animals (Supplementary Fig. 3). Inertial speed and variability were analysed following vehicle (V, 5–25 min following vehicle injection), ketamine (K, 5–25 min following ketamine injection), L-DOPA (LD, 65–85 min following L-DOPA injection) and L-DOPA followed by ketamine (LD → K, 5–25 min following ketamine injection) conditions/treatments (Fig. 2B–D).

Sub-anaesthetic ketamine is known to increase movement speed.^46,48,75^ In agreement with this, we found a significant effect of drug condition [Fig. 2C; two-way ANOVA: *F*(3,32) = 23.65, *P* < 0.001] and group [*F*(1,32) = 47.75, *P* < 0.001] on inertial speed, in addition to an interaction effect [*F*(3,32) = 29.97, *P* < 0.001], indicating that the effects of LD and LID → K on movement are enhanced in LID. Inertial speed in K and LD → K conditions was significantly higher compared with vehicle in sham and LID animals (*P* < 0.05). During dyskinesia in the LID animals, the inertial speed in the LD condition was significantly higher than in V and K conditions in LID and sham animals (*P* <0.001) and higher than LD in the sham animals (*P* < 0.001). We predicted that ketamine would reduce movement speed during dyskinesia given capacity of ketamine to reduce LAO-AIMs scores (Fig. 2A); however, no difference was observed between LD and LD → K (*P* = 0.4715). This suggests that the acute anti-dyskinetic effects of ketamine are from reduced LID-associated movements rather than movement speed.

As with inertial speed, there was a significant effect of drug condition [two-way ANOVA: *F*(3,32) = 23.31, *P* < 0.001] and group [*F*(1,32) = 36.75, *P* < 0.001] on inertial variability (Fig. 2D), in addition to an interaction effect [*F*(3,32) = 14.55, *P* < 0.001]. Ketamine during dyskinesia (LD → K) in the LID animals showed a significantly higher inertial variability compared with all other conditions in sham and LID animals (*P* < 0.05), suggesting that the effect of ketamine on LAO scores could result from increased variability in movement induced by ketamine.

### Ketamine eliminates LID-associated finely tuned gamma and increases low-gamma in M1

**Figure 3:**
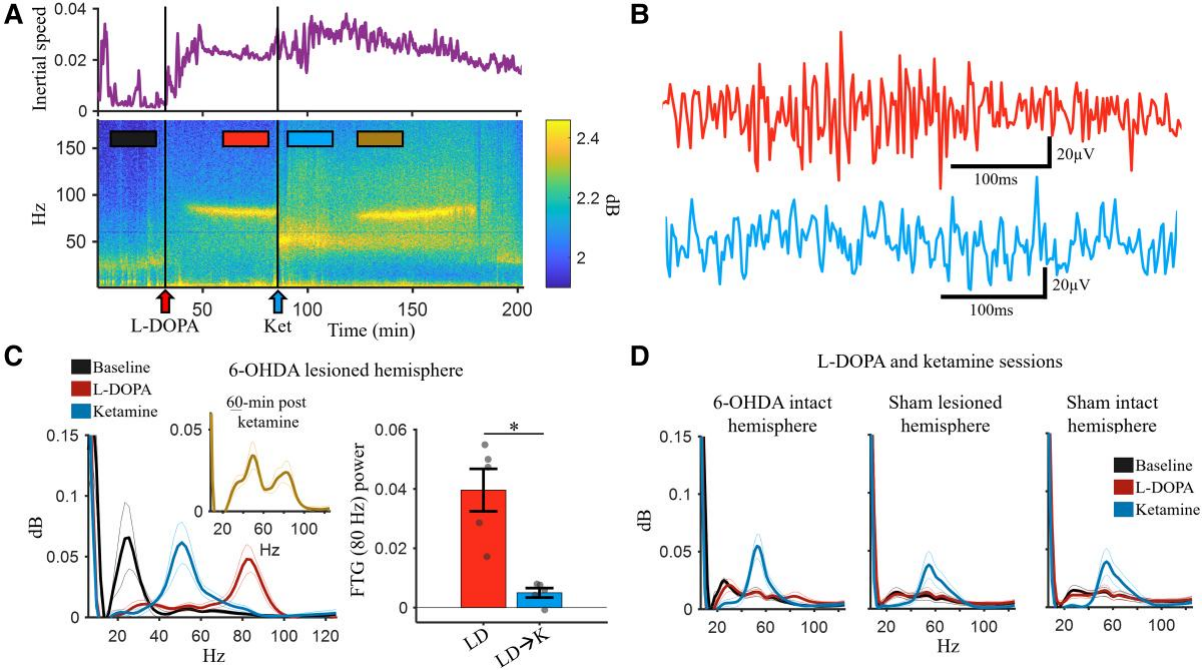
**Ketamine disrupts finely tuned gamma oscillations.** (**A**) Inertial speed (*top*) and spectrogram (*bottom*) for a representative L-DOPA followed by ketamine session for a single LID rat. The colour bars indicate 20 min analysis periods used in the rest of the figure. (**B**) Example local field potential traces during L-DOPA (*top*) and post-ketamine (*bottom*) periods indicated in **A**. (**C**) Power spectral density plot (*left*) showing the oscillatory component of the spectral response using Irregular-Resampling Auto-Spectral Analysis (IRASA, see ‘Materials and methods’ section) for the 6-hydroxydopamine (6-OHDA)-lesioned hemisphere during L-DOPA (LD) followed ketamine (K) sessions (*n* = 5, LID animals) for the 20 min periods indicated in **A**. *Inset*: Power spectral density for the 60 min period following ketamine injection (*rightmost* bar in **A**). *Right*: 80 Hz power (IRASA method) comparison between the LD and LD → K conditions in separate sessions (*n* = 5, LID animals). Following ketamine, the finely tuned gamma (FTG, ∼80 Hz) power reduced significantly compared with vehicle control (*P* = 0.0108, *t*-test). (**D**) Power spectral densities during the L-DOPA followed by ketamine sessions for the control hemispheres: 6-OHDA-intact hemisphere, sham-lesioned hemisphere and sham-intact hemisphere. Gamma (∼50 Hz) was seen during the post-ketamine period while the baseline and L-DOPA periods did not show the beta or FTG oscillations seen in the 6-OHDA-lesioned hemisphere in **B**. Outer lines show the 95% confidence intervals. *P < 0.05. Error bars show the mean ± standard error of the mean. LID = levodopa induced dyskinesia.

Consistent with previous research,^34^ we observed that administration of sub-anaesthetic ketamine during dyskinesia eliminated LID-associated FTG, which was replaced by low-gamma (Fig. 3A). Example local-field traces of ∼80 Hz FTG and ketamine-induced gamma are shown in Fig. 3B. Figure 3C shows the mean power spectral density plots averaged across all 6-OHDA-lesioned animals (*n* = 5) during sessions where L-DOPA was followed by ketamine. The power spectral densities were generated using the IRASA method,^63^ because this approach separates the oscillatory (shown) from the 1/*f* (fractal/aperiodic) component of the spectral signal.

Analysis of the power spectral densities revealed clear beta activity during the −25 to −5 min interval preceding drug injection (baseline; Fig. 3C), with beta oscillations being an established feature of dopamine depletion and PD.^76,77^ Beta was eliminated and replaced by FTG during the 35–55 min window following L-DOPA administration. FTG has long been associated with LID.^25,26,78^ Additionally, FTG power was strongly and positively correlated with LAO scores (Supplementary Fig. 3). For example, in sessions where L-DOPA was followed by vehicle, the correlation (Pearson’s *R*) between LAO scores and FTG power was 0.8517 (*n* = 5).

FTG (∼80 Hz) was eliminated and replaced with ∼50 Hz gamma during the 5–25 min window following ketamine injection (∼65 min following L-DOPA administration; Fig. 3C). Sub-anaesthetic ketamine is associated with gamma activity in the motor cortex,^48,75,79,80^ and previous work has shown that ketamine reduces FTG in animal models of LID.^34^ We showed that FTG power was significantly reduced in the LD → K treatment compared with LD (Fig. 3C, right; *P* = 0.0108, *t*-test). These effects were unique to the 6-OHDA-lesioned hemisphere, because neither FTG nor beta was observed in the 6-OHDA-intact hemisphere or sham animals (Fig. 3D).

There are at least two interpretations for the elimination of FTG following ketamine administration. It is possible that ketamine might induce an oscillatory state that out-competes or actively suppresses circuits involved in generating FTG. Alternatively, ketamine might simply shift the frequency response of circuits involved in FTG to the ∼50 Hz frequency associated with ketamine and locomotor activity.

Of further interest was the observation that although ∼80 Hz FTG re-emerged ∼60 min after ketamine administration (Fig. 3A), the ketamine-associated ∼50 Hz gamma remained, as indicated by the bi-modal power spectral density (Fig. 3C, inset). This indicates that FTG- and ketamine-induced gamma are not mutually exclusive, nor do they represent gain or frequency modulation of a single oscillator. Instead, this indicates that these two forms of gamma are generated by distinct oscillators.

### Beta-band activity in M1 is negatively correlated with movement in LID animals

**Figure 4:**
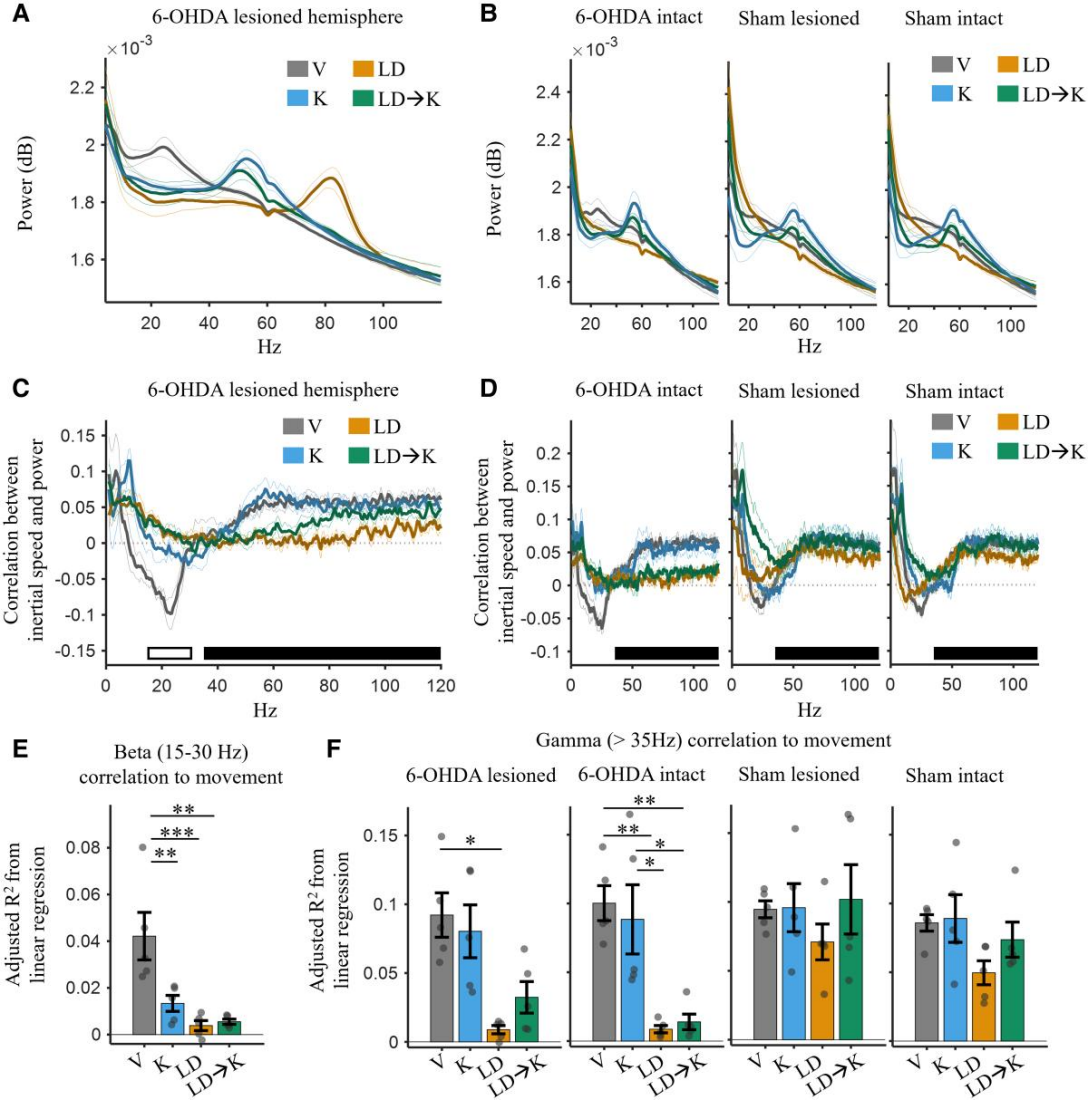
**Local-field correlation with inertial speed decreased during dyskinesia.** (**A**) Power spectral density plots for the 6-hydroxydopamine (6-OHDA)-lesioned hemisphere during the drug conditions shown in Fig. 2B. (**B**) Same as in **A**, but for the 6-OHDA-intact, sham-lesioned and sham-intact hemispheres. (**C**) Correlation (Pearson’s *R*) between power in frequencies ranging from 1 to 120 Hz and inertial speed in the 6-OHDA-lesioned hemisphere across animals (*n* = 5) for the four drug treatments in Fig. 2B. The bars indicate frequency ranges for beta (15–30 Hz, open bar) and gamma (35–120 Hz, solid bar) bands. Outer lines show the 95% confidence intervals. (**D**) Same as in **C** but for 6-OHDA-intact, sham-lesioned and sham-intact hemispheres. (**E**) The adjusted *R*^2^ from a general linear model, with the predictor being beta power and the outcome variable being inertial speed for the 6-OHDA-lesioned hemisphere across animals (*n* = 5). The levodopa (LD) and levodopa followed by ketamine (LD → K) conditions showed significantly lower *R*^2^ values compared with the vehicle (V) [*P* = 0.0007 and *P* = 0.0012, respectively, ANOVA, *F*(3,16) = 10.45, *P* = 0.0005], i.e. the negative correlation of beta power to inertial speed during vehicle was reduced following LD and LD → K. (**F**) Same as in **C** but with gamma-band frequency power as the predictor in the linear regression for the 6-OHDA-lesioned, 6-OHDA-intact, sham-lesioned and sham-intact hemispheres. Two-way ANOVA showed a significant effect of drug condition [*F*(3,64) = 14.97, *P* < 0.001] and hemisphere [*F*(3,64) = 6.47, *P* = 0.0007] on the regression, in addition to an interaction [*F*(9,64) = 2.05, *P* = 0.048]. *P < 0.05, **P < 0.01 and ***P < 0.001. Error bars show mean ± standard error of the mean.

Beta (15–30 Hz) in M1 is associated with immobility and motor dysfunction. Beta has been reported to be negatively correlated with movement onset and sustained movement,^81,82^ and increased beta power is a feature of PD.^77,83^ Consequently, we hypothesized that beta power would be negatively correlated with spontaneous movement in PD animals in the absence of L-DOPA. To investigate this, we correlated spectral activity at frequencies ranging from 1 to 120 Hz with inertial speed during vehicle, ketamine and LID conditions (Fig. 4).

Consistent with our prediction, beta was negatively correlated with movement during the vehicle condition in the 6-OHDA-lesioned hemisphere (Fig. 4C; for sham correlations, see Supplementary Fig. 5), where the *R* values were significantly negative in the 17–23 Hz range of beta (*t*-test, *P* < 0.05, Bonferroni–Holm correction). To quantify this relationship further, we used linear regression, with the predictor being beta power and the outcome variable being inertial speed. In the 6-OHDA-lesioned hemisphere, the adjusted *R*^2^ from the regression showed a significant decrease during the LD and LD → K conditions compared with the vehicle [Fig. 4E; *P*_LDvsV_ = 0.0007 and *P*_LD→KvsV_ = 0.0012, ANOVA: *F*(3,16) = 10.45, *P* = 0.0005]. There was also a significant decrease in the relationship between beta and movement following ketamine administration without L-DOPA (*P*_KvsV_ = 0.0094), which supports recent reports that ketamine can have anti-parkinsonian effects.^36^

### Gamma activity in M1 is not correlated with movement during LID

Wide-band gamma oscillations >30 Hz in M1 are positively associated with normal movement^84,85^ and sub-anaesthetic ketamine administration.^48,86,87^ In contrast, ∼80 Hz FTG in M1 is a neural signature of LID.^26,74^ Little is known about the relationship between normal and pathological forms of gamma and movement during LID or following ketamine administration. Given the relationship between FTG and LID, we hypothesized that FTG would be correlated positively with moment-to-moment changes in inertial speed during LID. Given the anti-dyskinetic effect of ketamine, we predicted that ketamine would enhance the correlation between M1 gamma and movement during LID.

Consistent with reports that M1 gamma is correlated with movement, a clear positive correlation was observed between all gamma frequencies and inertial speed in vehicle and ketamine conditions (Fig. 4C and D). Notably, and contrary to our original hypothesis, all gamma frequencies >35 Hz, including FTG, were not correlated with movement during LD or LD → K in the 6-OHDA-lesioned and intact hemispheres (Fig. 4C and D). This was particularly surprising given the strong FTG activity during LID (see Fig. 3A and C).

To quantify the overall relationship between gamma and movement, a linear regression was performed, with the predictors being power in each gamma-band frequency (35–120 Hz) and the outcome variable being inertial speed (Fig. 4F). There was a significant effect of drug condition [two-way ANOVA: *F*(3,64) = 14.97, *P* < 0.001] and hemisphere [*F*(3,64) = 6.47, *P* = 0.0007] on the *R*^2^ values, in addition to an interaction [*F*(9,64) = 2.05, *P* = 0.048]. The LD condition in the 6-OHDA-lesioned and 6-OHDA-intact hemispheres showed a significant decrease in adjusted *R*^2^ compared with vehicle across hemispheres (*P* < 0.05). Although the LD → K condition showed a significant decrease in the 6-OHDA-intact hemisphere compared with vehicle (*P* = 0.0067), this was not seen in the 6-OHDA-lesioned hemisphere (*P* = 0.2275). Thus, in the lesioned and intact hemispheres of LID animals, M1 gamma, including FTG, was negligibly correlated with inertial speed during LID, suggesting that M1 is functionally decoupled from head movements during LID.

### Correlation between single-unit activity in M1 and inertial movement is reduced during dyskinesia

**Figure 5:**
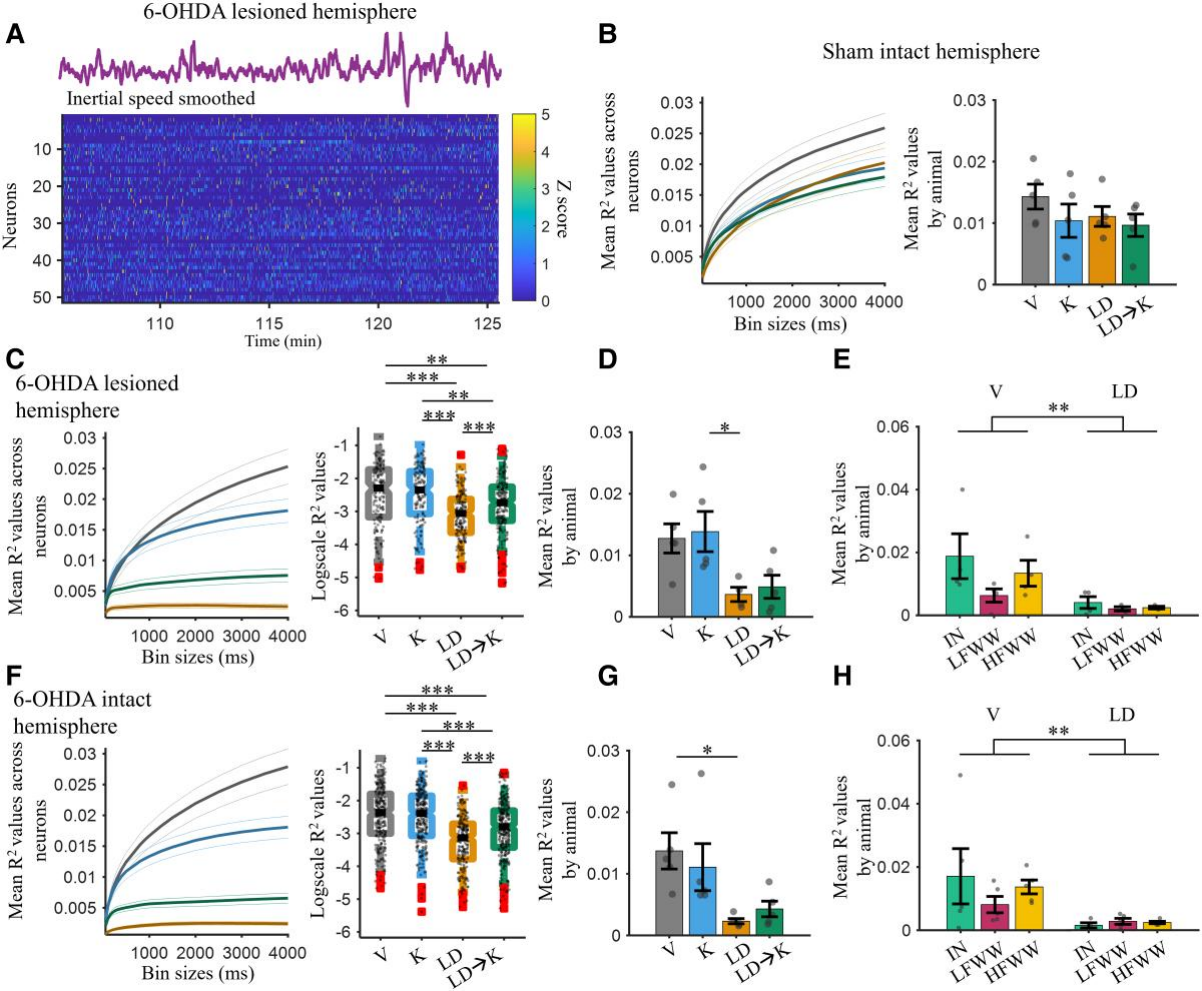
**Reduced M1 single-unit correlation with inertial speed during dyskinesia.** (**A**) Inertial speed (*top*) and *z*-scored firing rates of neurons binned at 500 ms (*bottom*) recorded from a single session in the 6-hydroxydopamine (6-OHDA)-lesioned hemisphere of one LID rat for a 20 min period during peak L-DOPA effect. (**B**) *Left*: Mean *R*^2^ values for single-unit correlation with inertial speed in the sham-intact hemisphere during the four drug treatments shown in Fig. 2B. Correlations were computed across several bin sizes ranging from 50 to 4000 ms. Outer lines show the 95% confidence intervals. *Right*: Mean *R*^2^ values averaged across all bin sizes and by animal (*n* = 5). The sham control hemisphere shows no difference between the drug treatments [ANOVA, *F*(3,16) = 0.86, *P* = 0.4824]. (**C**) Same as in **B** but for the 6-OHDA-lesioned hemisphere. *Right*: Logscale *R*^2^ values for all neurons averaged across bin sizes. The *R*^2^ value was lower in the levodopa (LD) and LD followed by ketamine (LD → K) conditions relative to vehicle [*P* < 0.001 and *P* < 0.0014, respectively, Kruskal–Wallis, χ^2^ (3728) = 78.98, *P* < 0.001]. (**D**) Mean *R*^2^ values averaged across all bin sizes and by animal (*n* = 5) for the 6-OHDA-lesioned hemisphere. There was a main effect for drug treatment [ANOVA, *F*(3,15) = 4.7, *P* = 0.0166], but the *post hoc* tests did not show a significant difference between treatments. (**E**) Mean *R*^2^ values by animal in the 6-OHDA-lesioned hemisphere separated by cell type: interneurons (IN), low-firing wide-waveform (LFWW) cells and high-firing wide-waveform (HFWW) cells. Only showing the vehicle and LD condition. (**F**–**H**) Same as **C**–**E** but for the 6-OHDA-intact hemisphere. The intact hemisphere showed similar effects of single-unit correlation with movement to the lesioned hemisphere in 6-OHDA animals. *P < 0.05, **P < 0.01 and ***P < 0.001. Error bars show the mean ± standard error of the mean. LID = levodopa-induced dyskinesia; V = vehicle.

Single-unit activity in M1 has been implicated in movement control and initiation,^11-13^ in motor learning^88-90^ and in LID.^91-93^ Here, we investigated the relationship between M1 single-unit activity and inertial speed during LID, ketamine and control conditions. We had no *a priori* hypothesis for the time scale of the relationship between M1 activity and movement. Consequently, a range of time scales was investigated by smoothing firing activity over intervals ranging from 50 to 4000 ms (see Materials and Methods). Figure 5A illustrates inertial speed and ensemble firing activity smoothed using a 500 ms window for a single session.

Given the unanticipated low correlation between local-field activity and movement during LID described above, we predicted that the correlation between M1 single-unit activity and movement would be reduced during LID. In agreement with our prediction, *R*^2^ values for neurons were significantly lower in LD and LD → K conditions relative to vehicle (Fig. 5C; *P* < 0.001 and *P* < 0.0014, respectively) in the 6-OHDA-lesioned hemisphere [Kruskal–Wallis: χ^2^(3728) = 78.98, *P* < 0.001]. At the animal level (Fig. 5D), there was a main effect of drug treatment [ANOVA: *F*(3,15) = 4.7, *P* = 0.0166]. *Post hoc* tests identified a significant difference only between the K and LD conditions (*P* = 0.0481). Interestingly, neurons in the intact hemisphere of the 6-OHDA rats also had significantly reduced *R*^2^ values in the LD condition [Fig. 5F; *P_LDvsV_* = 0, Kruskal–Wallis: χ^2^(31 091) = 142.69, *P* < 0.001] and when averaged by animal [Fig. 5G; *P*_LDvsV_ = 0.0245, ANOVA: *F*(3,16) = 4.7, *P* = 0.0155].

The same analysis described above was performed in sham animals, and no between-group differences were observed in either hemisphere at the animal level (Fig. 5B). When the sample comprised neurons, correlations were lower in the LD condition (Supplementary Fig. 6), suggesting that L-DOPA might reduce some neuron correlations with movement in healthy animals. Even so, this effect was larger in the 6-OHDA animals compared with sham (Supplementary Fig. 6F).

### No cell-type differences in the correlation between firing activity and inertial movement during LID

In the dyskinetic and PD states, previous work has identified cell-type-specific decrease in excitability^17,94^ and connectivity^8,93,95^ in M1. Thus, we investigated whether there were cell-type-specific differences in correlation with inertial speed during dyskinesia (Fig. 5E and H). The *R*^2^ values for the three cell types (IN, LFWW and HFWW) were compared between the V and LD conditions. ANOVA for the data from the lesioned hemisphere showed no effect of cell type [Fig. 5E; two-way ANOVA: *F*(2,16) = 1.7, *P* = 0.2149], a significant effect of drug treatment [*F*(1,16) = 9.38, *P* = 0.0074], and no interaction [*F*(2,16) = 0.9, *P* = 0.4266]. A similar effect of drug treatment alone was seen in the 6-OHDA-intact hemisphere [two-way ANOVA: *F*(1,23) = 10.64, *P* = 0.0034]. Thus, all cell types in the 6-OHDA animals showed a significant reduction in correlation with inertial speed during LID. Additionally, analysis of the correlation with movement by electrode depth did not identify any layer-specific differences (Supplementary Fig. 7).

### Ketamine activates a unique neural ensemble state in the 6-OHDA-lesioned hemisphere

At the population level, sub-anaesthetic ketamine alters interactions between neurons and neural ensemble dynamics.^38,96,97^ Ketamine might also induce an ‘activity switch’ in neurons, whereby ketamine reorganizes local cortical circuits by selectively activating or suppressing subpopulations of neurons.^96^ Furthermore, acute ketamine injection has also been shown to disrupt stability of spatial maps in the medial entorhinal cortex.^98^ How ketamine alters population dynamics under dopamine depletion and in dyskinetic conditions has not been investigated. In addition, little is known about how dyskinesia alters network-level dynamics in M1.

**Figure 6:**
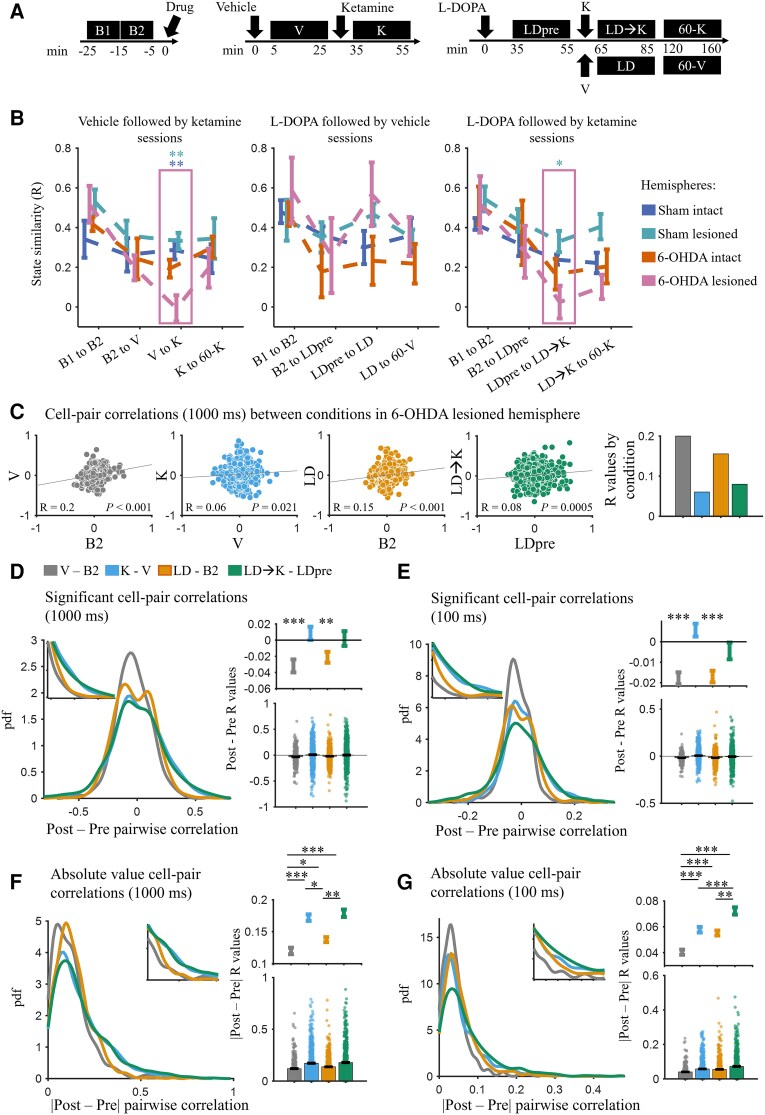
**Population state similarity and cell-pair correlations following drug treatments.** (**A**) Time periods used for state similarity and cell-pair correlation analysis. (**B**) Correlation between population states for the three session types: vehicle (V) followed by ketamine (K), L-DOPA (LD) followed by vehicle and L-DOPA followed by ketamine. Each state is a 10 min baseline or 20 min post-drug period, as shown in **A**. The LD → K and K states showed significant decrease in similarity between states compared with sham hemispheres [LDpre to LD → K: ANOVA, *F*(3,15) = 3.75, *P* = 0.0341; V to K: ANOVA, *F*(3,16) = 8.67, *P* = 0.0012]. (**C**) Scatter plots of cell-pair correlations (Pearson’s *R*) between pre- and post-drug treatments of interest. (**D**) Probability density function of the difference in pairwise correlations between the conditions shown in **C**. Only the significant cell-pair correlations were included, and the spike counts were binned at 1000 ms. *Right*: Difference *R* values for all neuron pairs. *Bottom*: All points. *Top*: Error bars showing the mean ± standard error of the mean (SEM) without points for better visualization of differences between drug conditions. The K and LD → K conditions did not show significant change in correlations, whereas LD and V showed significant decrease in correlations (*P* = 0.0016 and *P* < 0.001, respectively, Wilcoxon signed rank test). (**E**) Same as in **D** but for spike counts binned at 100 ms. As in the 1000 ms correlations, the K and LD → K conditions did not show significance, whereas LD and V conditions showed a significant decrease (*P* < 0.001, Wilcoxon signed rank test). (**F**) Same as in **D** but for absolute value of difference in cell-pair correlations. The K and LD → K conditions showed significantly higher magnitude of change compared with vehicle [*P* < 0.001, Kruskal–Wallis main-effect: χ^2^(32 168) = 48.62, *P* < 0.001] and L-DOPA periods (*P* = 0.0028 and *P* = 0.0107, respectively). (**G**) Same as in **F** but for spike counts binned at 100 ms. The K and LD → K conditions significantly increased the magnitude of change in correlations compared with vehicle [*P* < 0.001, Kruskal–Wallis, χ^2^(32 006) = 54.72, *P* < 0.001]. *P < 0.05, **P < 0.01 and ***P < 0.001. Error bars show the mean ± SEM.

To examine these issues, we computed a network-level measure of state similarity to quantify the extent to which the pattern of cell-pair correlations is similar between states (see Materials and Methods) for the states defined in Fig. 6A. A time scale of 1 s was chosen, because this time frame, when applied to single-unit activity, yielded clear correlations with inertial speed (Fig. 5C).

We observed that the similarity between the ketamine and vehicle ensemble states was lowest in the 6-OHDA-lesioned hemisphere [Fig. 6B, left; V to K: ANOVA, *F*(3,16) = 8.67, *P* = 0.0012]. Indeed, the similarity to vehicle approached zero, suggesting that ketamine induced a novel distribution of cell-pair correlations. A similar effect was observed during LID, where ketamine induced an ensemble state that differed considerably from LID [Fig. 6B, right; LDpre to LD → K: ANOVA, *F*(3,15) = 3.75, *P* = 0.0341]. These two results indicate that the capacity of ketamine to reorganize cell–cell interactions in M1 is largest in the dopamine-depleted hemisphere.

Changes in firing rate could potentially affect cell-pair correlations, which could, in turn, influence the state similarity findings. To address this, we compared firing rates during the drug treatments, which did not account for the changes we observed in state similarity measures (Supplementary Fig. 8).

To visualize the effect of ketamine and L-DOPA on ensemble states, scatterplots of cell-pair correlations were generated that compared correlations between control and drug treatments (Fig. 6C). In agreement with the preceding observation, correlations between states were lowest when ketamine was introduced. Specifically, correlations were low between the K and V states and the LD → K and LDpre states when compared with other conditions (Fig. 6C, right). It is interesting to note that despite L-DOPA profoundly changing behaviour in LID, the change in cell-pair interactions was relatively small compared with the ketamine-induced changes.

### Ketamine alters cell-pair correlations in a non-systematic manner

The changes in population-level activity described above might result from ketamine-induced alterations of the strength and direction of cell-pair interactions. This is suggested by recent work in a mouse model of schizophrenia showing that ketamine reduces cell-pair correlations measured using calcium imaging.^39^ Furthermore, ketamine can induce desynchronized states by altering excitability and spike entrainment to cortical gamma oscillations.^38^ Thus, we hypothesized that ketamine would decrease cell-pair correlations relative to vehicle or when given during LID in 6-OHDA-lesioned animals.

To investigate this, cell-pair correlations were computed at fast and slow time scales (100 and 1000 ms bin sizes) in each condition. The change in the correlation relative to the preceding state was computed (Fig. 6D and E). Correlations that were not significant (*P* > 0.05) were excluded from this calculation. Contrary to our hypothesis, there was no directional change in cell-pair correlations (a deviation from zero) following ketamine and LD → K conditions for the 1000 ms (Fig. 6D; *P* = 0.7035 and *P* = 0.9145, respectively, Wilcoxon signed rank with correction) and the 100 ms bin sizes (Fig. 6E; *P* = 0.4269 and *P* = 0.4269, respectively). Unexpectedly, correlations, on average, decreased following vehicle and L-DOPA administration. Specifically, correlations in LD decreased for the 1000 ms (*P* = 0.0047) and 100 ms (*P* < 0.001) bin sizes. This decrease in cell-pair correlations was also seen in the 6-OHDA-intact and sham hemispheres (Supplementary Fig. 9). Vehicle injection and L-DOPA injection in 6-OHDA animals were marked by large increases in movement, suggesting that motor activity in general reduces cell-pair correlations in M1 relative to periods of reduced activity.

The absence of a directional change in cell-pair correlations was unexpected following ketamine administration, given the observed reconfiguration of cell-pair correlations and ensemble states that followed ketamine injection (Fig. 6B). To investigate further how cell-pair correlations were changing following ketamine administration, we looked at the magnitude of change of the cell-pair correlations, regardless of the direction of change. This was measured as the absolute value of the difference in cell-pair correlations (Fig. 6F and G). In the 6-OHDA-lesioned hemisphere, the magnitude of change in cell-pair correlations for the 1000 ms bin size was significantly higher in K and LD → K conditions compared with vehicle [Fig. 6F; *P* < 0.001 for both, Kruskal–Wallis main effect: χ^2^(32 168) = 48.62, *P* < 0.001] and L-DOPA periods (*P*_KvsLD_ = 0.0030 and *P*_LD→KvsLD_ = 0.0118). Cell-pair correlations for the 100 ms bin size followed similar patterns, whereby the K and LD → K conditions significantly increased the magnitude of change compared with vehicle [Fig. 6G; *P*_KvsV_ < 0.001 and *P*_LD→KvsV_ = 0.0001, Kruskal–Wallis: χ^2^(32 006) = 54.72, *P* < 0.001]. Thus, ketamine produced deviant cell-pair correlations, where there was no directionality to the change but a large change in magnitude, indicating that correlations, on average, changed randomly, increasing and decreasing, following ketamine injection. This conclusion is not definitive, because this effect was not significant at the animal level (Supplementary Fig. 10).

## Discussion

Uncontrolled movements are a defining and debilitating feature of LID. The neural generators of these movements are not understood, and treatments are limited. Although multiple regions in the cortico-basal ganglia–thalamic loop are implicated in LID, considerable evidence indicates the involvement of the motor cortex.^15,17,26,92^ Here, we investigated the relationship between M1 single-unit and local-field activity and LID-associated movements measured with standard LAO-AIMs scoring and at high temporal resolution using head-mounted inertial sensors. Furthermore, we investigated whether sub-anaesthetic ketamine restores dysregulated neural activity associated with LID, given the evidence that ketamine reduces LID acutely and for weeks following administration.^33,34,36^ To summarize, we found that the relationship between M1 single-unit and local-field activity and movement becomes profoundly disrupted in LID, and this relationship is partly restored by ketamine in M1 neurons. We also found that ketamine significantly and selectively alters M1 ensemble state in the dyskinetic hemisphere by generating distinct patterns of cell–cell interactions and by increasing the variance of the distribution of cell-pair correlations.

### The correlation between M1 local-field and single-unit activity with inertial speed is reduced during LID

In sham animals and in vehicle conditions in 6-OHDA-lesioned animals, changes in single-unit and gamma-band activity were robustly correlated with ongoing inertial movement at sub-second to second scales (Figs 4 and 5). Furthermore, a strong negative correlation between beta-band activity and movement was observed in the parkinsonian rats prior to L-DOPA administration. Beta has long been associated with immobility, and increased beta is a signature of PD.^77,83^ To our knowledge, this is the first time that a negative correlation between beta (specifically in the 17–23 Hz range of beta) and inertial movement has been identified and shown to be reduced following L-DOPA or ketamine administration.

Regarding LID, L-DOPA administration to the 6-OHDA animals eliminated the relationship between gamma and movement (Fig. 4), and correlations between M1 single-unit activity and movement fell below control conditions (Fig. 5). There is a parallel between these findings in LID and reports of the loss of specificity of neural responses to movement in PD.^22,99,100^ These results are interesting, because decoupling was stronger during LID relative to the dopamine-depleted state, and the effect was observed in M1. Future work could investigate whether striatal neurons also express reduced responsiveness to movement during LID. Another interesting observation was that the decoupling of inertial movement to ongoing neural activity was observed in the lesioned and intact hemispheres of LID animals, suggesting that chronic dopamine depletion in one hemisphere reorganizes inter-hemispheric circuits.^92,101^ Future studies could investigate inter-hemispheric communication, sub-second changes in dopamine release, and their relationship to movement changes in the intact hemisphere.

Taken together, these observations suggest that the relationship between M1 activity and movement changes considerably during LID, with M1 becoming effectively decoupled from sub-second changes in movement. Although human and preclinical studies have correlated the FTG oscillations in M1 with the onset and offset of dyskinetic movements and LID severity,^26,74,102^ this does not reflect the modulation of M1 neurons or local-field activity to continuous and ongoing movements during LID. In fact, the emergence of FTG could indicate the decoupling in M1 activity, leading to the dyskinetic state.

Considerable anatomical, physiological and behavioural evidence supports M1 involvement in LID.^15,92,103^ Even so, the precise role played by M1 in generating dyskinetic movements is not understood. Decoupling of single-unit and local-field activity to movement during dyskinesia suggests that dysregulated M1 activity does not directly activate specific dyskinetic movements. Instead, decoupling might indicate that M1 loses its capacity to organize or constrain activity in downstream pattern generators in the brainstem, spinal cord or cerebellum.^104^ Lack of regulation could free these generators to initiate dyskinetic movements. This is a possibility, because subcortical generators are capable of producing complex orofacial, locomotor and dyskinetic movements.^104,105^ These ideas might relate to the proposed role of M1 in inhibiting competing and inappropriate actions.^106-109^ Indeed, many, if not most, M1 efferent projections target inhibitory neurons in the brainstem and spinal cord.^107,110,111^ Furthermore, centre-surround inhibition has been reported following M1 stimulation.^106^ Thus, aberrant movements in LID might not be initiated directly by M1, but might be initiated indirectly, by way of reduced M1 inhibition of competing movements.

#### Ketamine partly restores the correlation between M1 activity and movement in LID

Consistent with previous reports, ketamine acutely reduced LAO severity (Fig. 2A) and FTG power (Fig. 3C) during LID.^34^ Sub-anaesthetic ketamine is also pro-kinetic in rodents and increases gamma-band activity correlated with locomotor activity.^48,86,87^ Given these observations, we expected that ketamine would restore the correlation between movement and neural activity during LID.

Ketamine administration increased M1 neuron correlation with inertial speed during LID (Fig. 5). However, this rescue of correlation in single-unit activity was significantly lower compared with vehicle and the effect of ketamine in a non-dyskinetic state. Thus, there was a partial rescue of M1 neural correlation with movement following ketamine administration. A complete reversal of the LID-induced decoupling with movement back to baseline levels would theoretically induce pro-parkinsonian effects. Therefore, the partial increase in correlation with movement following ketamine could indicate a unique state induced by ketamine that both alleviates LID symptoms and preserves the anti-parkinsonian effects. This hypothesis was not tested directly in our analysis, thus future work could examine the exact nature of the states induced by PD, LID and ketamine as a function of correlation with movement in M1 activity.

Interestingly, although the highly selective NMDA receptor antagonist MK-801 by itself is both anti-dyskinetic and pro-parkinsonian,^112^ the combination of MK-801 and a μ- and δ-opioid receptor agonist is both anti-dyskinetic and anti-parkinsonian.^113^ In addition, blocking opioid receptors has differential effects on the anti-dyskinetic and anti-parkinsonian activity of ketamine.^114^ Given the action of ketamine as an opioid receptor agonist,^115^ perhaps a mix of NMDA and opioid receptor activity by ketamine is involved in inducing this state on a molecular level, separating ketamine from selective NMDA receptor antagonists.

### Ketamine does not systematically enhance or reduce cell-pair correlations but increases their variability

Ketamine profoundly impacted the pattern of cell-pair interactions in M1, and this effect was largest in the dopamine-depleted hemisphere (Fig. 6B). Although it is known that ketamine induces unique patterns of neuronal activity,^39,43,96,98^ it is notable that capacity of ketamine to re-organize cell-pair correlations observed here was most prominent in the depleted hemisphere during LID. The ability of ketamine to reconfigure local and global network dynamics is thought to underlie its effectiveness in treating mood disorders by prompting state transitions.^116,117^

The non-directional reconfiguration of cell-pair correlations suggests a mechanism whereby ketamine initially alters ongoing network-level activity, followed by ketamine-induced increases in brain-derived neurotrophic factor^118-120^ to produce lasting alterations in activity and connectivity.^42,117,121,122^ This is supported further by a study showing that the long-term anti-dyskinetic activity of ketamine is dependent on brain-derived neurotrophic factor signalling and can be blocked completely by a tropomyosin receptor kinase B (TrkB) antagonist.^36^

### Limitations

A limitation of this study is the lack of data from female rats. Unfortunately, extending the experimental design to sample female rats fully was beyond the scope of the present study. Investigating LID in female rats is an important future direction. A further limitation is that the movement kinematics obtained from the inertial measurement data are limited to head movements. Thus, our correlation analysis with movement is not specific to occurrences of canonical behavioural movements during LID, such as abnormal contralateral limb movements.

## Supplementary Material

awae386_Supplementary_Data

## Data Availability

Derived data supporting the findings of this study are available from the corresponding author on request.
