# Supplementary material for: Decoupling of motor cortex to movement in Parkinson’s dyskinesia rescued by sub-anaesthetic ketamine
Source: Brain. 2024 Nov 25;148(6):2135–50. doi: 10.1093/brain/awae386 (PMC12129741; doi:10.1093/brain/awae386)
Supplement: awae386_Supplementary_Data [file awae386_supplementary_data.pdf]

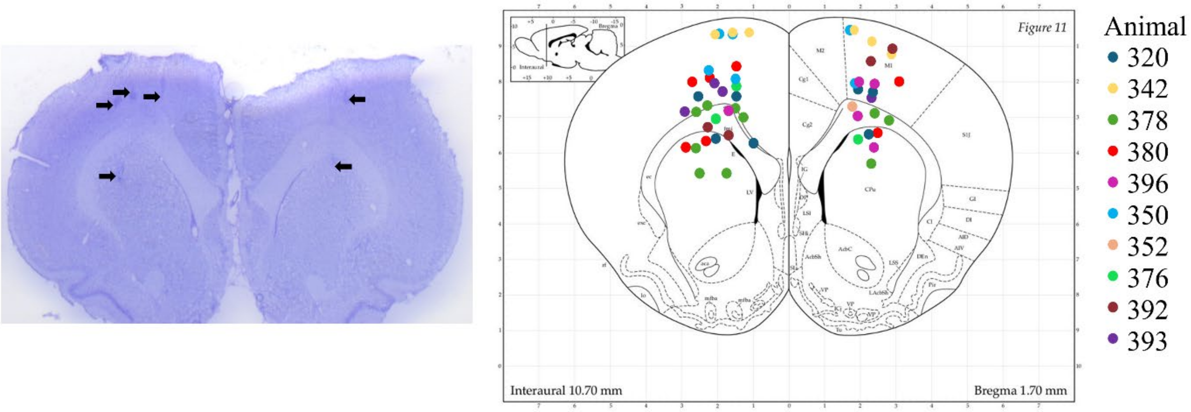

Supplementary Figure 1: Verification of tetrode location. LEFT: Cresyl-violet stained section from one rat. Arrows point to tetrode location at the end of the experiment. RIGHT: Location of tetrode end points by rat. The variation in AP coordinates was <1 mm. The number of dots per animal is the location of the tetrodes clearly visible from histology.

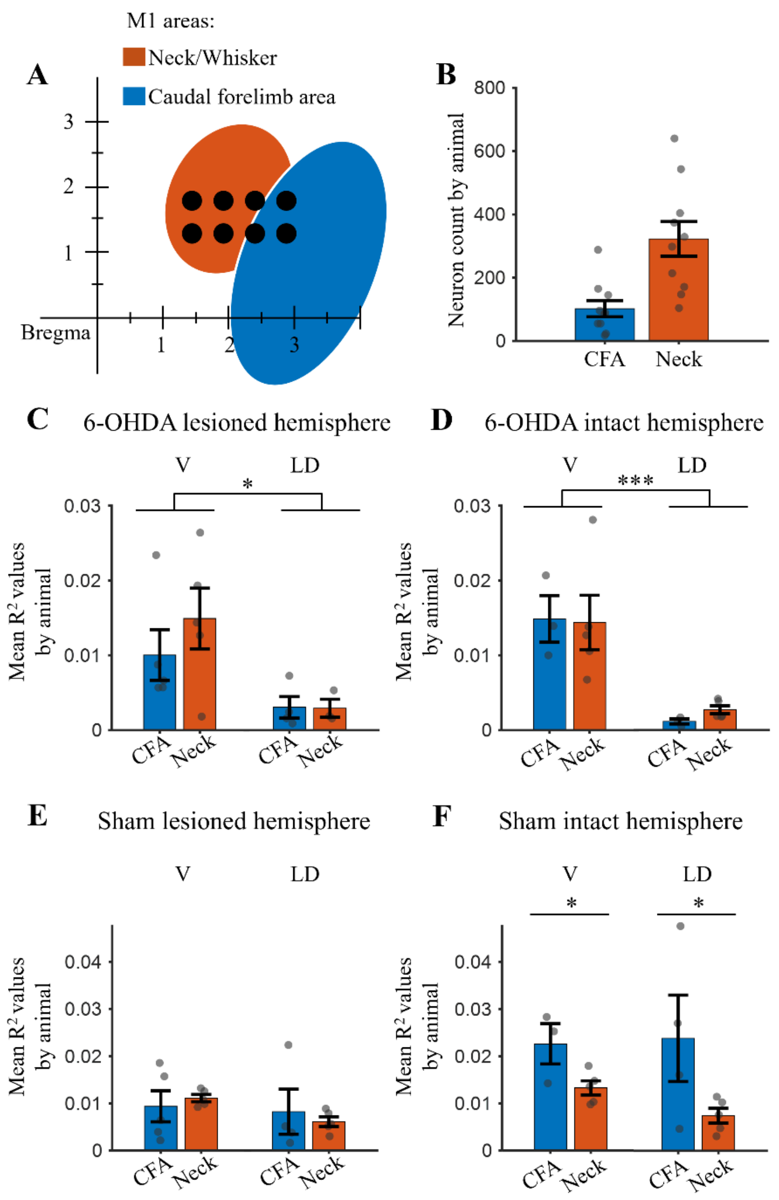

Supplementary Figure 2: Somatotopic differentiated M1 single-unit correlation to inertial speed during dyskinesia. (A) Schematic of tetrode locations on the simplified somatotopic organization of M1[1,2]. **(B)** Neuron counts in the neck/whisker and caudal forelimb regions by animal (n = 10). **(C)** Mean R^2 values for single-unit correlation to inertial speed averaged across all bin sizes and by animal (n = 5) in the 6-OHDA lesioned hemisphere for the vehicle and L-DOPA conditions. The two-way ANOVA revealed a significant effect of drug condition ($F(1,13)$ = 8.0648, P = 0.0139) but no effect of M1 area ($F(1,13)$ = 0.5074, P = 0.4888). **(D)** Same as (C) but for the 6-OHDA intact hemisphere. The two-way ANOVA revealed a significant effect of drug condition ($F(1,12)$ = 22.009, P = 0.0005) but no effect of M1 area ($F(1,12)$ = 0.04, P = 0.8447). **(E)** Same as (C) but for the sham lesioned hemisphere. The two-way ANOVA revealed no effect

of drug condition ($F(1,15) = 1.27$, $P = 0.2768$) or M1 area ($F(1,15) = 0$, $P = 0.9469$). **(F)** Same as (C) but for the sham intact hemisphere. The two-way ANOVA revealed no effect of drug condition ($F(1,13) = 0.24$, $P = 0.6357$) but a significant effect of M1 area ($F(1,13) = 7.24$, $P = 0.0185$). However, there was no interaction effect ($F(1,13) = 0.54$, $P = 0.4739$). * $P < 0.05$, ** $P < 0.01$, and *** $P < 0.001$. Error bars show mean ±SEM.

1. Brown, A. R., & Teskey, G. C. (2014). Motor Cortex Is Functionally Organized as a Set of Spatially Distinct Representations for Complex Movements. Journal of Neuroscience, 34(41), 13574–13585. https://doi.org/10.1523/JNEUROSCI.2500-14.2014

2. Sekiguchi, Y., Muramatsu, K., Tamaki, T., Ikutomo, M., & Kurosawa, K. (2019). Neck and trunk representations in the primary motor cortex in rats. *Journal of Physical Therapy Science*, *31*(8), 608–611. https://doi.org/10.1589/jpts.31.608

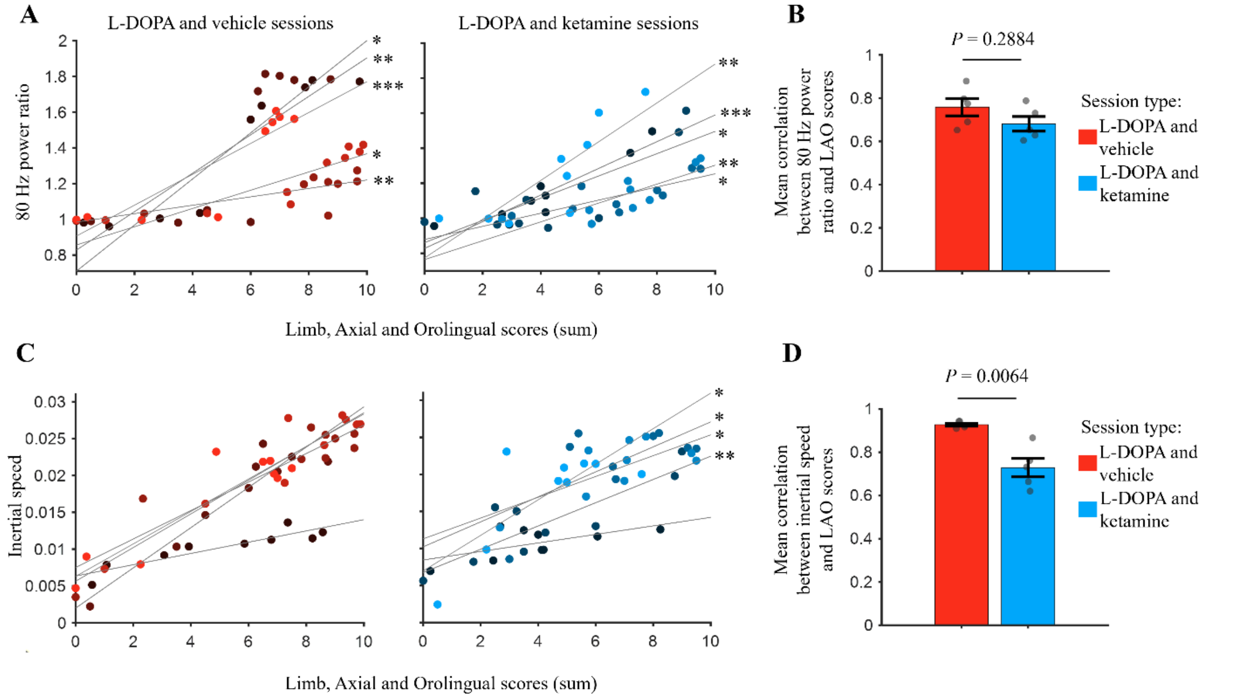

Supplementary Figure 3: Finely-tuned ~80 Hz gamma oscillations were positively correlated with dyskinesia severity. (A) Scatter plots showing correlation between 80 Hz power ratio and LAO scores for 6-OHDA lesioned hemisphere of LID animals (n = 5) during L-DOPA followed by vehicle (left) and L-DOPA followed by ketamine (right) sessions. To obtain the 80 Hz power ratio, the power in the 74 to 94 Hz band (calculated using IRASA method) was divided by the mean power in adjacent frequency bands (65 to 70 Hz and 100 to 105 Hz). There was a significant positive correlation between 80 Hz power and LAO scores for all animals (n = 5) in both sessions. **(B)** Mean correlation (Pearson's R) during the sessions in A. Both sessions showed large positive correlations and there was no significant difference (P = 0.2884, T-test) in R values between the two session types. * $P < 0.05$, ** $P < 0.01$, and *** $P < 0.001$. Error bars show mean ±SEM. **(C)** Scatter plots showing correlation between inertial speed and LAO scores for 6-OHDA lesioned hemisphere of LID animals (n = 5) during L-DOPA followed by vehicle (left) and L-DOPA followed by ketamine (right) sessions. There was a significant positive correlation between inertial speed and LAO scores for all animals (n = 5, p < .001 for each animal) in L-DOPA followed by vehicle sessions. There was a significant positive correlation between inertial speed and LAO scores in four out of five animals in the L-DOPA followed by ketamine sessions. **(D)** Mean correlation (Pearson's R) during the sessions in C. Both sessions showed large positive correlations and there was a significant difference (P = 0.0064, T-test) in R values between the two session types. * $P < 0.05$, ** $P < 0.01$, and *** $P < 0.001$. Error bars show mean ±SEM.

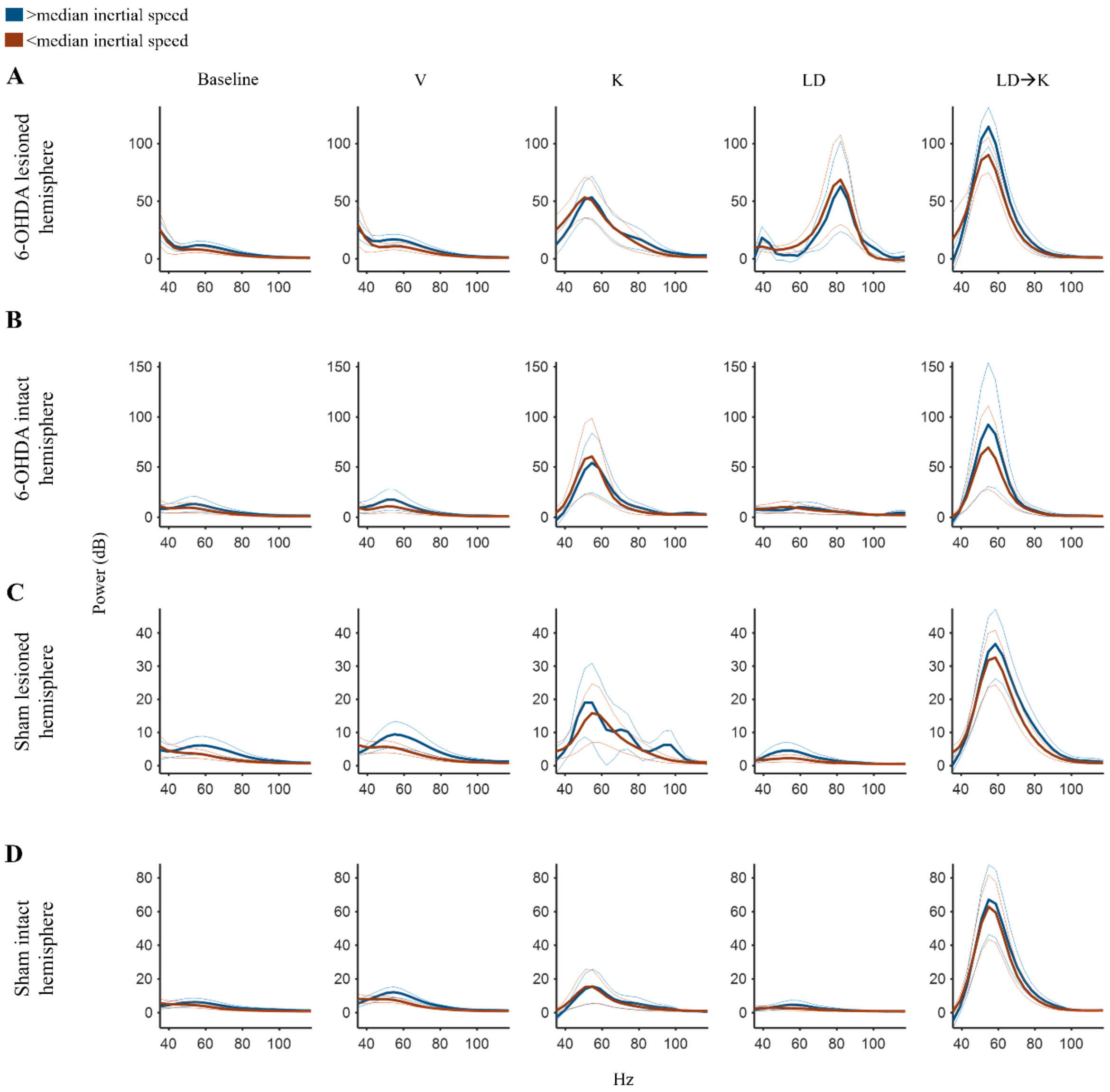

Supplementary Figure 4: Movement-induced power spectral density plots for gamma frequencies. (A) Power spectral density plots (IRASA, 35-120 Hz) for the 6-OHDA lesioned hemisphere by animal (*n* = 5) during drug conditions detailed in Fig. 2B. Baseline (-5 to -25 min prior to drug administration), V = vehicle (5 to 25 min following vehicle injection), K = ketamine (5 to 25 min following ketamine injection), LD = L-DOPA (65 to 85 min following L-DOPA injection), LD→K = L-DOPA followed by ketamine (5 to 25 min following ketamine injection). The PSDs are segmented into low and high movement periods (>200 ms) for the 20-minute drug period using a median split of the inertial speed data. **(B-D)** Same as (A) but for the 6-OHDA intact hemisphere, sham lesioned hemisphere, and sham intact hemisphere. Error bars show mean ±SEM.

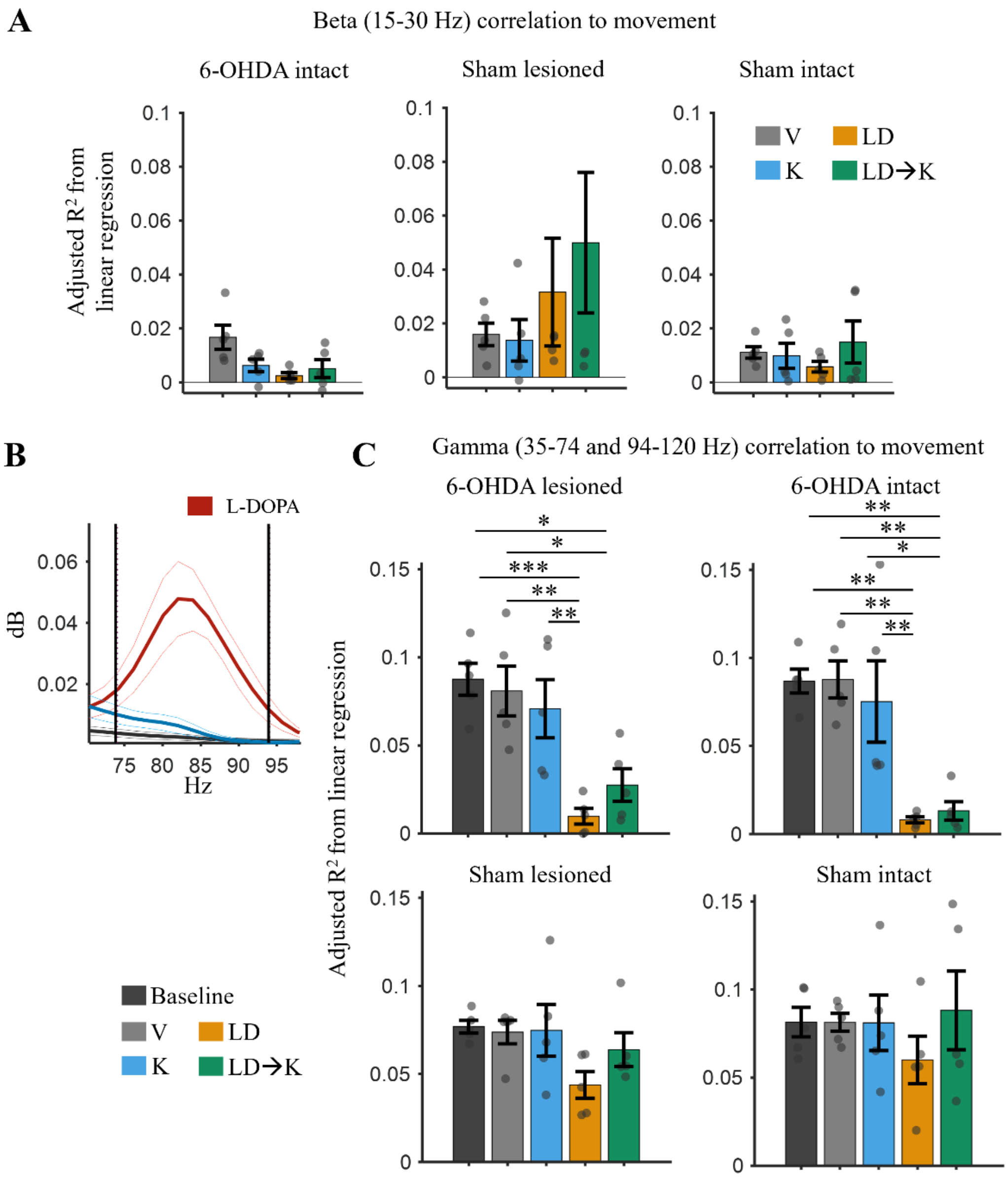

Supplementary Figure 5: Beta and gamma correlation to inertial speed. (A) The error bars show adjusted R^2 from a general linear model with the predictor being beta power and the outcome variable being inertial speed for the 6-OHDA intact, sham lesioned, and sham intact hemispheres across animals (n = 5). No significant changes in beta correlation to movement was observed between drug treatments (detailed in Fig. 2B). **(B)** Power density plot (IRASA) showing the frequencies (74-94 Hz) for the finely-tuned gamma band oscillations. **(C)** The error bars show adjusted R^2 from a general linear model with the predictor being gamma power (35-74 and 94-120 Hz) excluding the finely tuned gamma range (74-94 Hz) and the outcome variable being inertial speed for the 6-OHDA lesioned, 6-OHDA intact, sham lesioned, and sham intact hemispheres across animals (n = 5). The baseline period (-5 to -25 min prior to drug administration) correlation

is also shown in addition to the drug conditions (detailed in Fig. 2B). The removal of finely tuned 80 Hz gamma power did not change the decrease in gamma correlation to movement during LD and LD→K conditions in the 6-OHDA lesioned and 6-OHDA intact hemispheres. * $P < 0.05$, ** $P < 0.01$, and *** $P < 0.001$. Error bars show mean ±SEM.

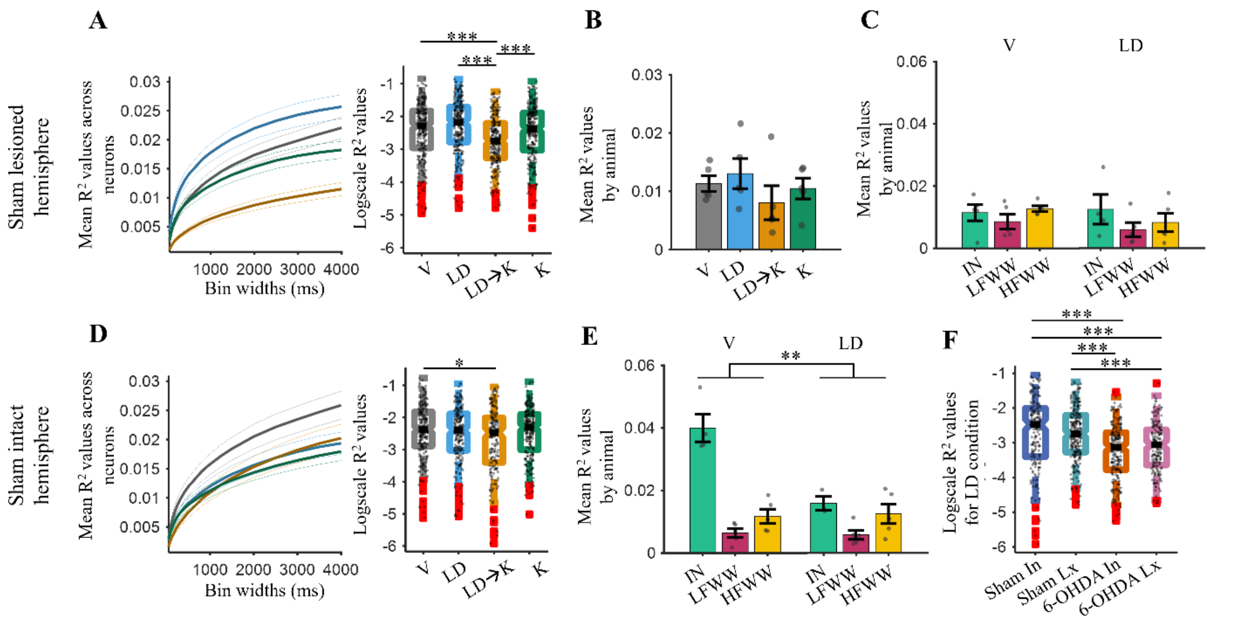

Supplementary Figure 6: M1 single-unit correlation to inertial speed in sham animals. (A) LEFT: Mean R^2 values for single-unit correlation to inertial speed in the sham lesioned hemisphere during the four drug treatments shown in Fig 2B. Correlations were computed across several bin sizes ranging from 50 to 4000 ms. Outer lines show the 95% confidence intervals. RIGHT: Logscale R^2 values for all neurons averaged across bin sizes. The R^2 values was lower in the LD condition relative to vehicle, ketamine, and LD→K ($P < 0.001$ for all, Kruskal-Wallis main-effect: Chi-square(3,1112) = 52.77, $P < 0.001$). **(B)** Mean R^2 values averaged across all bin sizes and by animal ($n = 5$). No significant main effect of drug treatment (ANOVA, $F(3,16) = 0.86$, $P = 0.4824$)**.** **(C)** Mean R^2 values by animal in the sham lesioned hemisphere separated by cell-type: Interneurons (IN), low firing wide waveform (LFWW) cells, and high firing wide waveform (HFWW) cells. Only showing the vehicle and LD condition. **(D)** Same as in A. but for sham intact hemisphere. The R^2 values was lower in the LD condition relative to vehicle ($P = 0.0159$ for all, Kruskal-Wallis main-effect: Chi-square(3, 927) = 9.66, $P = 0.0217$). **(E)** Same as in C. but for sham intact hemisphere. There was a significant main effect of cell-type (Two-way ANOVA: $F(2,21) = 32.28$, $P = 0$), drug treatment ($F(1,21) = 13.62$, $P = 0.0014$), and an interaction effect ($F(2,21) = 12.11$, $P = 0.0003$). This however was due to the increased correlation of interneurons following vehicle injection and the consequent increase in firing and movement. **(F)** Logscale R^2 values for all neurons compared across hemispheres during the LD condition. There was significant main effect of group (Kruskal-Wallis: Chi-square(3,874) = 71.73, $P < 0.001$), where the 6-OHDA lesioned and 6-OHDA intact hemispheres showed a significantly larger decrease in movement correlation following LD treatment than the sham lesioned and sham intact hemispheres. * $P < 0.05$, ** $P < 0.01$, and *** $P < 0.001$. Error bars show mean ±SEM.

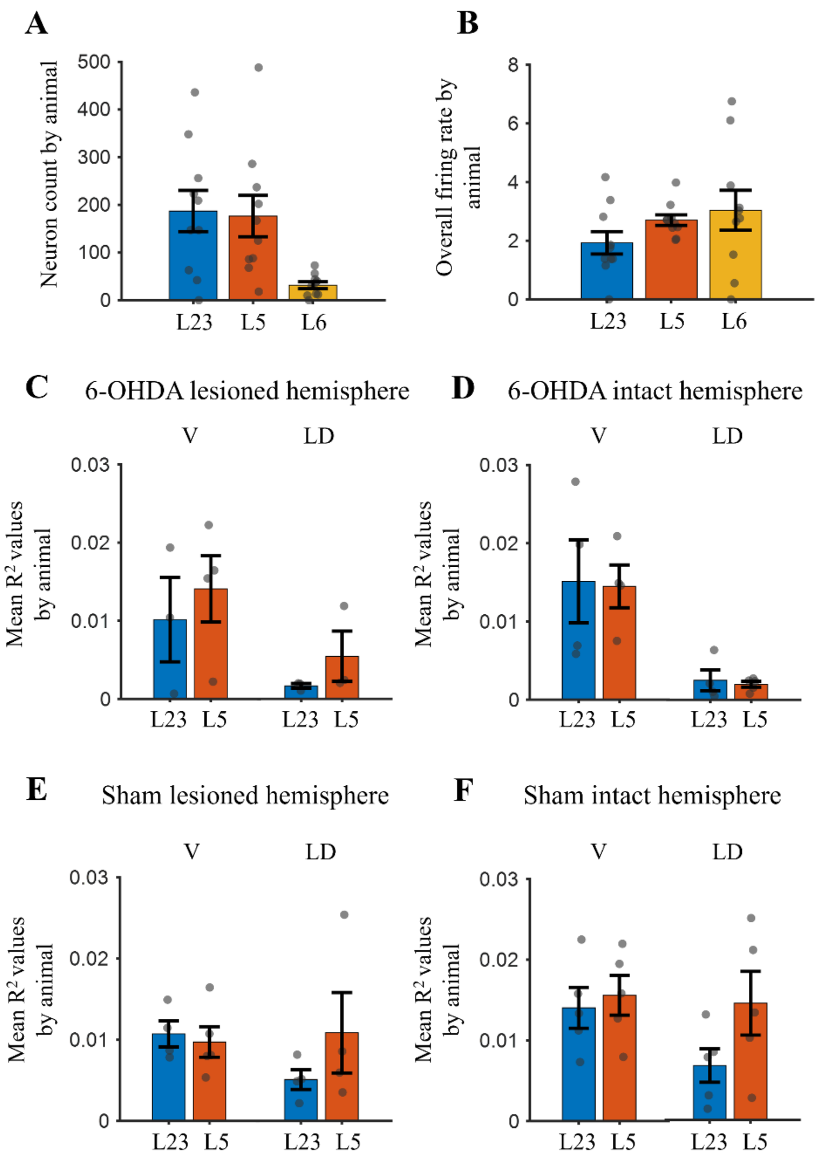

Supplementary Figure 7: Layer differentiated M1 single-unit correlation to inertial speed during dyskinesia. (A) Neuron counts in layer 2/3, 5 and 6 of M1 by animal (n = 10). **(B)** Firing rate of neurons by animal (n = 10) and layer. **(C)** Mean R^2 values for single-unit correlation to inertial speed averaged across all bin sizes and by animal (n = 5) in the 6-OHDA lesioned hemisphere for the vehicle and L-DOPA conditions. The two-way ANOVA revealed no effect of drug condition ($F(1,9) = 4.6627$, $P = 0.0591$) or M1 layer ($F(1,9) = 0.9483$, $P = 0.3555$). **(D)** Same as (C) but for the 6-OHDA intact hemisphere. The two-way ANOVA revealed a significant effect of drug condition ($F(1,13) = 19.1719$, $P = 0.0007$) but no effect of M1 layer ($F(1,13) = 0.0403$, $P = 0.8439$). **(E)** Same as (C) but for the sham lesioned hemisphere. The two-way ANOVA revealed no effect of drug condition ($F(1,13) = 0.7501$, $P = 0.4021$) or M1 layer ($F(1,13) = 0.6683$, $P =$

0.4283). **(F)** Same as (C) but for the sham intact hemisphere. The two-way ANOVA revealed no effect of drug condition ($F(1,16) = 2.1893$, $P = 0.1583$) or M1 layer ($F(1,16) = 2.6495$, $P = 0.1231$). * $P < 0.05$, ** $P < 0.01$, and *** $P < 0.001$. Error bars show mean ±SEM.

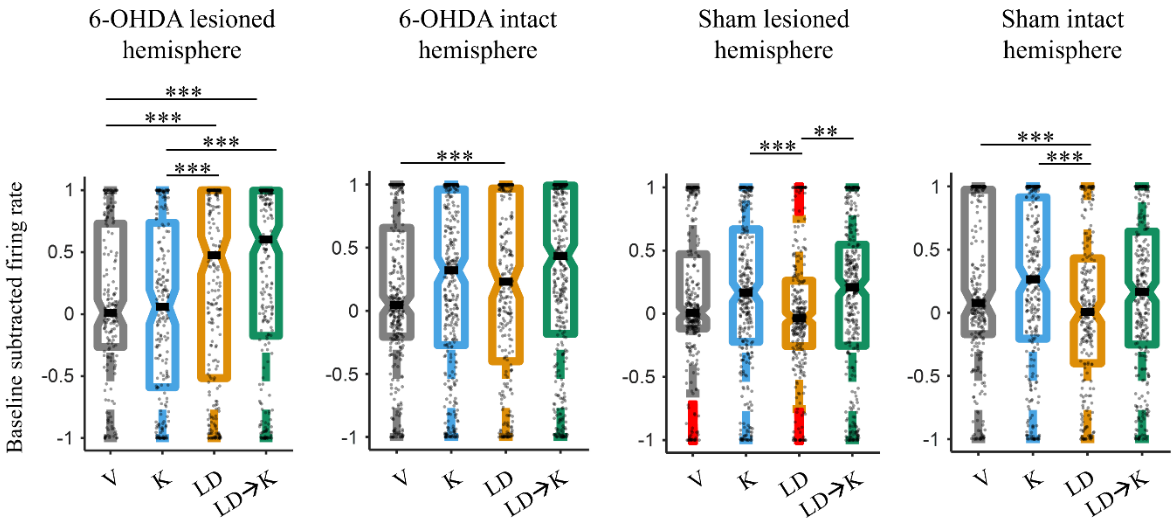

Supplementary Figure 8: Firing rate changes of M1 neurons during drug treatments. Baseline subtracted firing rates for the 20-minute drug treatment/condition periods shown in Fig. 2B for the 6-OHDA lesioned, 6-OHDA intact, Sham lesioned, and Sham intact hemispheres. Firing rates were standardized (-1 and 1) using (drug condition firing rate – baseline firing rate)/(drug condition firing rate – baseline firing rate). Comparisons were made using Kruskal-Wallis test of variance and Dunn-Sidak post hoc correction. * $P < 0.05$, ** $P < 0.01$, and *** $P < 0.001$.

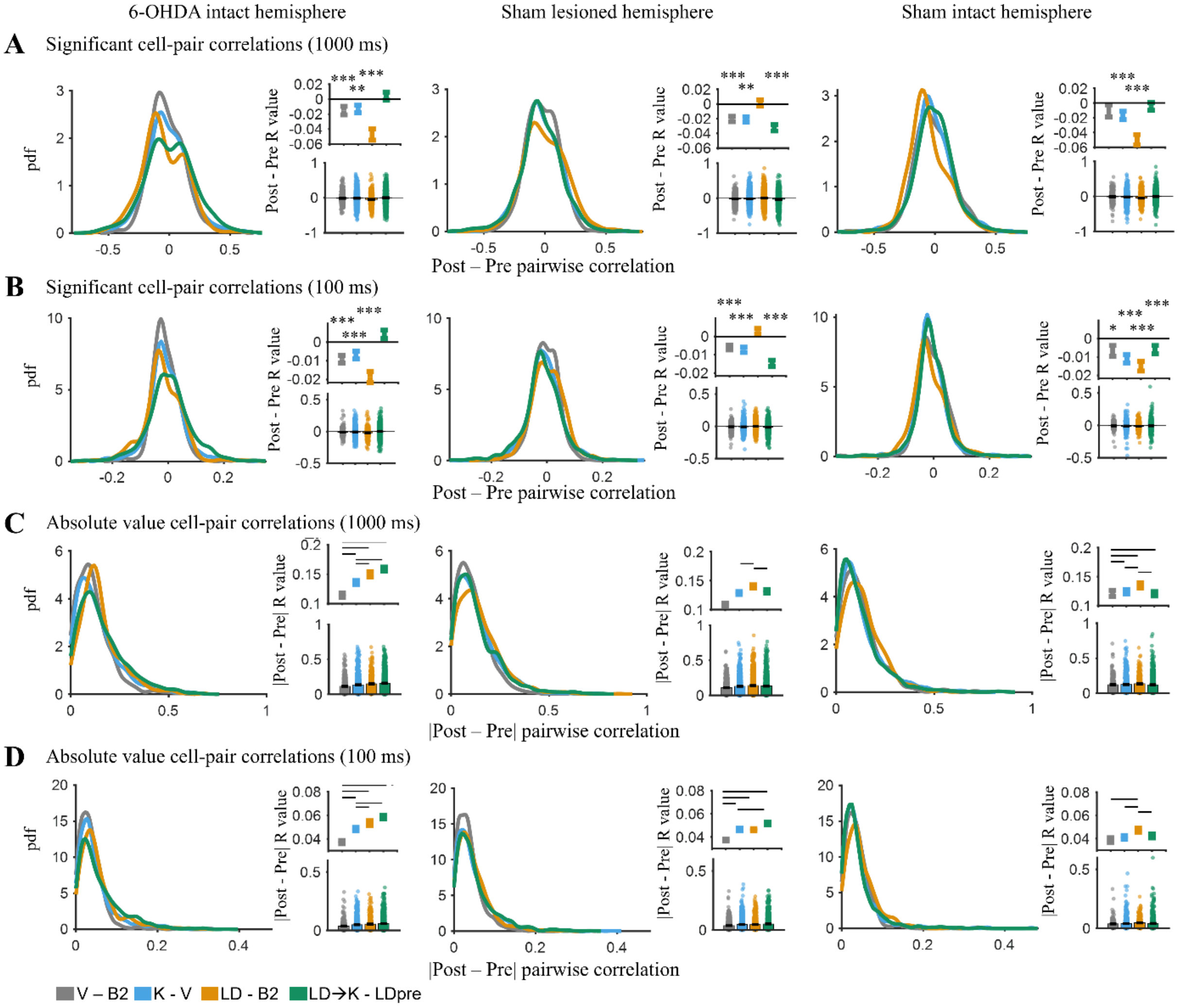

Supplementary Figure 9: Cell-pair correlations for control hemispheres. (A) Probability density function of the difference in pairwise correlations between the conditions shown in Fig. 6D, for the 6-OHDA intact, sham lesioned, and sham intact hemispheres. Mean Post – Pre R values were calculated for each condition: Error bars showing all data points (bottom) and error bars with mean ±SEM (top) for better visualization. **(B)** Same as in A. but for spikes binned at 100 ms. Wilcoxon sign rank test with Bonferroni-Holm correction. **(C)** Same as in A. but for the absolute value of the difference in pairwise correlations. **(D)** Same as in C. but for spikes binned at 100 ms. Kruskal-Wallis test of variance and Dunn-Sidak post hoc correction. * $P < 0.05$, ** $P < 0.01$, and *** $P < 0.001$. Error bars show mean ±SEM.

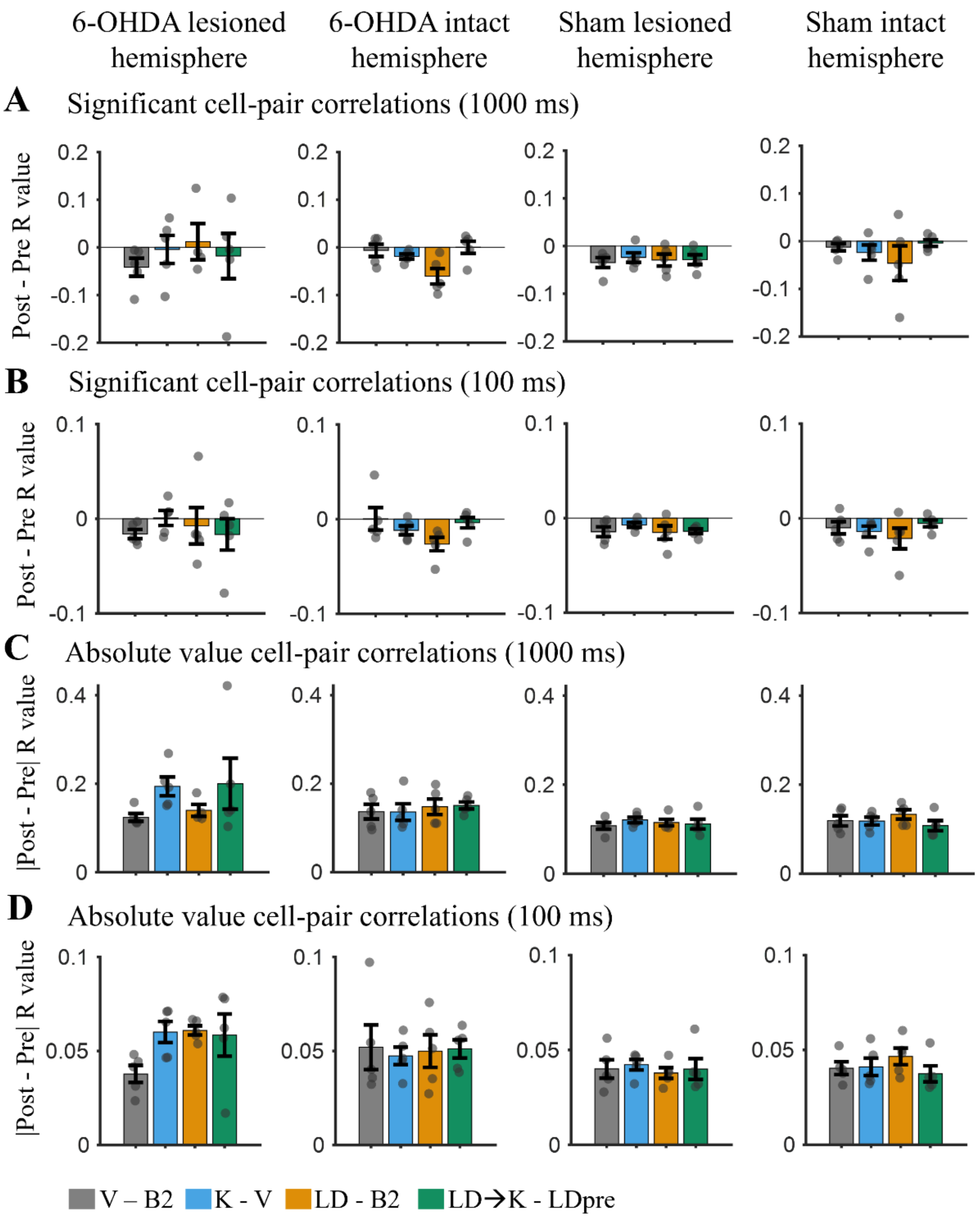

Supplementary Figure 10: Cell-pair correlations by animal. (A) Error bars showing mean difference in pairwise correlations averaged by animal between the conditions shown in Fig. 6D, for the 6-OHDA lesioned, 6-OHDA intact, sham lesioned, and sham intact hemispheres. **(B)** Same as in A. but for spikes binned at 100 ms. **(C)** Same as in A. but for the absolute value of the difference in pairwise correlations. **(D)** Same as in C. but for spikes binned at 100 ms. ANOVA revealed no significant differences between drug conditions. Error bars show mean ±SEM.
